# Methodology for contamination detection and reduction in fermentation processes using machine learning

**DOI:** 10.1007/s00449-025-03194-6

**Published:** 2025-06-26

**Authors:** Xuan Dung James Nguyen, Y. A. Liu, Christopher C. McDowell, Luke Dooley

**Affiliations:** 1 Aspen Tech Center of Excellence in Process System Engineering, Department of Chemical Engineering, Virginia Polytechnic Institute and State University, Blacksburg, VA 24061 USA; 2 Novonesis Biological, Inc., 5400 Corporate Circle, Salem, VA 24153 USA

**Keywords:** Contamination, Fermentation processes, Machine learning, SHAP feature importance, Hyperparameter optimization

## Abstract

This paper demonstrates an accurate and efficient methodology for fermentation contamination detection and reduction using two machine learning (ML) methods, including one-class support vector machine and autoencoders. We also optimize as many hyperparameters as possible prior to the training of the ML models to improve the model accuracy and efficiency, and choose a Python platform called Optuna, to enable the parallel execution of hyperparameter optimization (HPO). We recommend using Bayesian optimization with hyperband algorithm to carry out HPO. Results show that we can predict contaminated fermentation batches with recall up to 1.0 without sacrificing the precision and specificity of non-contaminated batches, which read up to 0.96 and 0.99, respectively. One-class support vector machine outperforms autoencoders in terms of precision and specificity even though they both achieve an outstanding recall of 1.0. These models demonstrate high accuracy in detecting contamination without requiring labeled contaminated data and are suitable for integration into real-time fermentation monitoring systems with minimal latency and retraining needs. In addition, we benchmark our ML methods against a traditional threshold-based contamination detection approach (mean $\pm$ 3 $\upsigma$ rule) to quantify the added value of using data-driven models. Finally, we identify important independent variables contributing to the contaminated batches and give recommendations on how to regulate them to reduce the likelihood of contamination.

**Supplementary Information:**

The online version contains supplementary material available at 10.1007/s00449-025-03194-6.

## Introduction

### Machine learning models for contamination detection

Machine learning (ML) has found several applications in detecting contaminations in a variety of applications, such as food and medical packaging industries [1, 2] and contamination risks in drinking water [3, 4]. For fermentation, there have not been any reported studies involving proper applications of ML models for both contamination detection and reduction. Some early papers consider some simple artificial neural networks (ANNs) [5] and fuzzy neural networks [6]. More recent uses of ML methods in contamination detection in fermentation include Heese et al. [7] and Wu et al. [3] using Deep Neural Networks (DNNs). A careful review of the limited number of prior studies reveals that they do not really emphasize the importance of recall in contamination detection, do not include detailed HPO prior to training the ML models to improve model performance, and fail to identify the contribution of independent variables and their desired changes to reduce contamination. This paper addresses all these aspects plus additional key issues, as outlined in the next subsection.

### Contributions and significance

Our contributions are as follows:

1. Our study differentiates from previous studies of fermentation contamination detection by demonstrating two accurate and efficient ML methods to detect anomalies, including autoencoders and one-class support vector machines.
2. We emphasize F2-score optimization without too much sacrifice on precision. Recognizing the critical importance of recall in contamination detection, we adopt the F2-score as the primary evaluation metric and tune model hyperparameters accordingly to prioritize minimizing false negatives, while considering steps to do so without sacrificing too much precision and specificity (correctly predict non-contaminated batches).
3. We present a comprehensive comparative analysis: The proposed framework is benchmarked across diverse datasets and evaluated against important metrics such as precision, recall, specificity, F2-score, Receiver Operating Characteristic—Area Under the Curve (ROC-AUC), and Precision-Recall—Area Under the Curve (PR-AUC), providing insights into the trade-offs between methods.
4. Hyperparameter optimization (HPO) with Bayesian optimization with hyperband (BOHB): We systematically outline the step-by-step methodology, present practical insights for hyperparameter tuning using BOHB from a state-of-the-art HPO package on Python called Optuna, and provide the relevant Python codes in Sections S2 to S7 in the Supporting Information to demonstrate thoroughly how to utilize Optuna for HPO.
5. In industrial settings, contamination detection often relies on simple threshold-based rules applied to process variables. However, such methods lack the flexibility to capture complex, multivariate, and temporal relationships. In this work, we compare our ML-based models with a conventional threshold-based baseline to demonstrate the advantages of data-driven approaches in both detection accuracy and robustness.
6. Real-world applicability: This study highlights the proposed framework’s practical utility by demonstrating its application to real fermentation process datasets, showcasing its efficiency and effectiveness in detecting fermentation contamination events, and identifying important process variables contributing to contamination, hence giving recommendations on how to reduce the risk of contamination. In addition to evaluating model performance, this study discusses critical practical considerations such as concept drift, retraining frequency, and latency constraints, which are essential for implementing ML models in production-scale fermentation processes.

## Methodology

### Dataset and data preprocessing

Our dataset contains 246 batches of fermentation data obtained by Novonesis Biological Inc. in Salem, Virginia, with a total of 23 labeled contaminated batches and 223 healthy batches. Each batch lasts for a different time duration, with a starting time and an end time. There are several different variables of interest in each batch, and each variable is collected with different timestamps. Therefore, the amount and the type of variables of interest are different among different batches, and in each batch, the amount of data points for each variable is also different. There are also several missing and invalid values, including those of numeric, categorical, and date-time forms. As industrially available data can often have these inconsistencies, these industrial fermentation data offer an opportunity to develop methodologies to deal with real datasets. Therefore, data preprocessing and data manipulation are crucial. There are several key steps implemented in the data preprocessing step:

- Drop empty rows/columns;
- Drop unnecessary/unuseful rows/columns;
- Convert all potential numeric columns to valid numeric values and then drop all those invalid numeric values;
- Identify timestamp columns for each variable, remove invalid timestamp columns, and convert timestamps to valid date-time formats;
- Find the timestamp column with the most valid values for each batch;
- Sort each batch dataset using the timestamp column with the most valid values to ensure proper alignment, and each dataset is in chronologic order;
- Handle duplicated timestamp values using their mean values;
- Setting the timestamp column with the most valid values as the index, ready to be resampled;
- Resample each dataset to a uniform 5-s interval and fill in missing values using the linear interpolation method and the forward fill;
- Remove any empty rows/columns post-resampling;
- Replace the “Fermenter Time, hr” column with values of hours, starting from 0 for the earliest timestamp value.

After preprocessing, each batch with valid data is saved in an Excel file, ready for the data manipulation or feature engineering step. An important purpose of this step is to extract meaningful “features” from each batch, mainly statistical summaries, rolling window calculations, and lag-based features. These include: (1) aggregated statistical features such as mean, standard deviation (std), min, and max values; (2) rolling features such as the rolling mean over a window of five timestamp values; and (3) lag features such as 1-step lagged values. In addition, after calculating the rolling mean and lagged values, we also compute the previous summary statistics for the 5-step rolling mean and the 1-lagged time values. The resulting information is invaluable because, in time-series datasets, different types of features capture critical process variations, deviations, and trends that may indicate contamination. The “engineered features” generated in these steps give useful insights into process dynamics, variability, and potential contamination causes. Below is a more detailed breakdown of why the statistically engineered features are important for this task:

1. Static aggregated statistics (mean, std, min, and max values):
2. Mean: represents the central tendency of the variable throughout the batch. That means a significant shift in mean values may indicate contamination
3. Standard deviation (std): measures variability within a batch. Higher variations may indicate contamination due to unexpected fluctuations (e.g., microbial growth affecting pH or dissolved oxygen)
4. Min and max values: capture extreme values. A spike in temperature, pressure, or dissolved oxygen beyond expected limits can be a contamination indicator
5. Rolling features (5-step moving average statistics)
6. Capture process stability: a sudden change in rolling means can indicate contamination
7. Reduce noises: since fermentation data are highly dynamic, rolling features filter short-term fluctuations and emphasize trends
8. Help in early anomaly detection since contaminants can cause gradual drifts in parameters, which are easily detected via rolling statistics.
9. Lag features (1-step time shift)
10. Detect delayed contamination effects: since some contaminants cause gradual deviations
11. Help identify delays: since some variables like temperature, pH, or oxygen level respond slowly to contamination, lag features can track this time-based dependency
12. Useful for predictive models: because machine learning models can learn from past values to predict potential contamination before it fully manifests.

By extracting meaningful features, machine learning models and statistical analysis can distinguish contaminated batches from normal batches effectively, enabling early detection, root-cause analysis, and process optimization. After applying the feature engineering step, a single dataset is generated, with each column representing each meaningful feature for each batch, and with each row representing data for each batch with their corresponding contamination labels, which is 0 for normal batches and 1 for contaminated batches. This dataset after feature engineering includes a total of 267 columns with three columns labeled “Batch_ID”, “Batch_Names”, and “Contamination Label”, corresponding to 264 engineered features and 264 rows corresponding to 264 batches. Then, this dataset is ready to be used as input for the following ML methods of choice.

### Selection of ML models for fermentation contamination detection (anomaly detection)

We propose to use two different ML methods to develop predictive models for contamination detection in fermentation, including one-class support vector machine (OCSVM) and autoencoders (AE). Since the labeled contamination data are scarce in this case, our contamination detection task is essentially anomaly detection. Therefore, we use unsupervised machine learning methods like OCSVM and AE are used. These models train only on normal batches (hence the unsupervised methods and also the name “one-class”) and flag out abnormal batches. Details on the mathematics and how these two models perform the training and detection are as follow.

#### AutoEncoder (AE)

AutoEncoders (AEs) [8] are unsupervised neural networks that learn a low-dimensional representation of normal data and reconstruct it. The reconstruction error is used as an anomaly score. A deep encoder consists of mainly three types of layers:

1. Encoder layer, which compresses input data into a latent space;
2. Bottleneck layer which captures the essential features of normal batch behavior;
3. Decoder layer, reconstruct the input from the latent space.
  1 $$\widehat{\text{x}}={\text{f}}_{\uptheta }\left(\text{x}\right)$$

where $x$ is the original input, $\widehat{x}$ is the reconstructed input, ${f}_{\theta }$ is the autoencoder function parametrized by $\theta$. Since the purpose of the AE is to reconstruct the inputs, its inputs and outputs have the same shape. The AE structure used in this paper is fully connected feedforward deep neural networks (FCFFDNNs).

The model is trained only on normal batches (hence the unsupervised method), and 10% of the normal data are used for validation while training the AE. This follows because an AE will learn the typical patterns of normal batches, and when a contaminated (anomalous) batch is passed through, it is not reconstructed well, leading to higher reconstruction errors. This reconstruction error is typically the mean squared error (MSE) between the input and the output, which is used for anomaly detection:

2 $$\text{MSE}=\frac{1}{\text{n}}\sum_{\text{i}=1}^{\text{n}}{\left({\text{y}}_{\text{i}}-\widehat{{\text{y}}_{\text{i}}}\right)}^{2}$$

where ${x}_{i}$ and ${\widehat{x}}_{i}$ are the observed and corresponding predicted values of the i^th^ measurements, and n is the total number of observations. Batches with reconstruction errors above a threshold determined using percentiles are flagged as contaminated.

For this particular dataset used in this study, the architecture of our AE consists of:

- Input layer: Size 264, corresponding to engineered features from fermentation batch data.
- Encoder: Several dense layers with ReLU activations, followed by batch normalization layers, dropout layers, and L1/L2 regularization to prevent overfitting and encourage sparsity.
- Bottleneck layer: A dense layer with reduced dimensionality representing the compressed latent space capturing the essential patterns of normal data.
- Decoder: A mirror of the encoder, reconstructing the input from the bottleneck representation.

Many hyperparameters such as numbers of dense layers between the encoder and the decoder, number of neurons per layer, L1 and L2 regularization rates, dropout rate, learning rate, batch size, and types of optimizer are tuned using BOHB from Optuna (more details provided in Sections S1 in the Supporting Information). The model is trained using the MSE loss function between inputs and reconstructed outputs. During inference, batches are flagged as contaminated if their reconstruction errors exceed a threshold determined by percentile analysis on non-contaminated validation data.

#### One-class support vector machine (OCSVM)

One-class support vector machine [9], proposed by Schölkopf et al. [10], is also an unsupervised ML method that learns a boundary around normal batches and flags deviation as contaminated:

3 $$\text{f}\left(\text{x}\right)={\upomega }^{\text{T}}.\upphi \left(\text{x}\right)-\uprho$$

where $\phi \left(x\right)$ is the transformation that maps the input $x$ to a higher-dimensional space; $\omega$ is a weight vector; and $\rho$ is a bias term that controls the decision boundary. The optimization problem for OCSVM can be formulated as:

4 $$\begin{array}{*{20}c} {{\text{min}}} \\ {\omega ,\xi _{{\text{i}}} ,\rho } \\ \end{array} \frac{1}{2}\left\| \omega \right\|^{2} + \frac{1}{{\nu {\text{n}}}}\sum\limits_{{{\text{i}} = 1}}^{{\text{n}}} {\xi _{{\text{i}}} } - \rho$$

subject to:

5 $${\upomega }^{\text{T}}\upphi \left({\text{x}}_{\text{i}}\right)\ge\uprho -{\upxi }_{\text{i}}$$

where $\nu$ controls the proportion of outliers and support vectors in the training data and ${\xi }_{i}$ are slack variables that allow for some misclassification. Since real-world contamination data are often non-linearly separable, we use the kernel trick to map it into a higher-dimensional space, where it becomes separable. Some common kernels are listed in Table 1 [11].

**Table 1:** Common kernel functions used for transformation in OCSVM

Common kernel functions	Mathematical forms
Radial Basis Function (RBF) Kernel	${\text{K}}\left( {{\text{x}}_{{\text{i}}} ,{\text{x}}_{{\text{j}}} } \right) = {\text{exp}}\left( { - \alpha\parallel {\text{x}}_{{\text{i}}} - {\text{x}}_{{\text{j}}}\parallel^{2} } \right)$ (6)
Linear Kernel	${\text{K}}\left( {{\text{x}}_{{\text{i}}} ,{\text{x}}_{{\text{j}}} } \right) = {\text{x}}_{{\text{i}}}^{{\text{T}}} {\text{x}}_{{\text{j}}}$ (7)
Polynomial Kernel	${\text{K}}\left( {{\text{x}}_{{\text{i}}} ,{\text{x}}_{{\text{j}}} } \right) = \left( {\beta {\text{x}}_{{\text{i}}}^{{\text{T}}} {\text{x}}_{{\text{j}}} + {\text{c}}} \right)^{{\text{d}}}$ (8)
Sigmoid Kernel	${\text{K}}\left( {{\text{x}}_{{\text{i}}} ,{\text{x}}_{{\text{j}}} } \right) = {\text{tanh}}\left( {\gamma {\text{x}}_{{\text{i}}}^{{\text{T}}} {\text{x}}_{{\text{j}}} + {\text{c}}} \right)$ (9)

The model is also trained only on normal batches (unsupervised method), hence the name “one-class”. Unlike standard SVMs, which classify between two or more classes, the goal of OCSVM is to learn the distribution of normal data and then construct a hyperplane that encloses the majority of normal points, flagging outliers that deviate significantly as contaminated. Once trained, the decision function of the OCSVM classifies a new sample *x* as normal or contaminated as follows:

10 $$\text{f}\left(\text{x}\right)=\sum_{\text{i}=1}^{\text{n}}{\alpha }_{\text{i}}\text{K}\left({\text{x}}_{\text{i}},{\text{x}}_{\text{j}}\right)-\rho$$

where ${\alpha }_{\text{i}}$ are Lagrange multipliers obtained during training, and $\uprho$ is the threshold that indicates anomalies. For this contamination task, if $\text{f}\left(\text{x}\right)\ge 0$, the sample is normal, and if $\text{f}\left(\text{x}\right)<0,$ it is contaminated.

The OCSVM model defines a boundary that encompasses the majority of the normal (non-contaminated) data in a high-dimensional space. Any new batch falling outside this boundary is considered an anomaly. It is particularly useful in cases where contaminated examples are rare or unavailable for training. The key components and considerations include:

- Kernel functions: We evaluate several kernel types including Radial Basis Function (RBF), polynomial, and sigmoid kernels, with the RBF kernel ultimately providing the best trade-off in flexibility and performance.
- Hyperparameters: The $\upupsilon$ (nu) parameter controls the expected proportion of outliers; $\upgamma$ (gamma) controls the kernel’s influence radius; tol defines convergence criteria. All are optimized via BOHB.

The decision function evaluates the distance of each sample to the separating hyperplane. Samples with negative decision values are flagged as anomalies. This method offers interpretability and strong performance for modeling complex boundary conditions in high-dimensional feature space.

Together, these models provide complementary approaches to unsupervised contamination detection: AE captures reconstruction difficulty from nonlinear embeddings, while OCSVM focuses on boundary violations in transformed feature space.

### F2-Score and performance metrics

The performance of each ML model is evaluated by recall (sensitivity), Eq. (11); precision, Eq. (12); and specificity, (Eq. (13), for contaminated samples.

11 $$Recall (Sensitivity)= \frac{True Positives (TP)}{True Positives \left(TP\right)+False Negatives (FN)}$$

12 $$Precision= \frac{True Positives (TP)}{True Positives \left(TP\right)+False Positives (FP)}$$

13 $$Specificity= \frac{True Negatives (TN)}{True Negatives \left(TN\right)+False Positives (FP)}$$

In addition, the Area Under the Curve (AUC) of the Receiver Operating Characteristics (ROC) plot and the AUC of the Precision–Recall (PR) are also measured. The ROC curve demonstrates how well a binary classifier can distinguish between two groups across different threshold settings. It is plotted by comparing the true positive rate (sensitivity) against the false positive rate (1-specificity). The ROC-AUC quantifies the model’s ability to separate the classes, with values closer to 1 indicating stronger classification performance. However, in this case, since the dataset is highly imbalanced due to rare contaminated batches, the ROC-AUC can be misleading as a model can achieve high ROC-AUC by simply predicting the majority (non-contaminated) class. Therefore, PR-AUC is more important, with higher values indicating that the model balances recall and precision well, effectively detecting contamination (high recalls) with fewer false alarms (high precisions).

Moreover, in this particular scenario, our primary objective is to identify as many contaminated (anomalous) batches as possible because failure to detect contamination can have serious consequences. That means that false negative (FN) cases (that is, missed contamination cases) are much worse than false positive (FP) (flagging normal batches as contaminated). Therefore, recall should be prioritized to be increased compared to increasing precision. The F $\beta$-score is a weighted harmonic mean of precision and recall, formulated as:

14 $$F\beta =\left(1+{\beta }^{2}\right)\times \frac{precision\times recall}{\left({\beta }^{2}\times precision\right)+recall}$$

Therefore, F1-score and F2-score are formulated as

15 $$F1=\left(1+{1}^{2}\right)\times \frac{precision\times recall}{\left({1}^{2}\times precision\right)+recall}=\frac{2\times precision\times recall}{precision+recall}$$

16 $$F2=\left(1+{2}^{2}\right)\times \frac{precision\times recall}{\left({2}^{2}\times precision\right)+recall}=\frac{5\times precision\times recall}{4\times precision+recall}$$

From Eqs. (15) and (16), we see that recall is twice as important in F2-score compared to F1-score. As a result, we use the F2-score as another performance metric and the objective to be maximized in HPO, instead of the F1-score. However, the purpose of HPO is still trying to find the best performance model, which in this case means optimizing the F2-score without sacrificing too much precision and specificity, meaning trying to predict as many contaminated batches as possible while ensuring precision and specificity as high as they can possibly be.

### General implementation flowchart

**Fig. 1:**
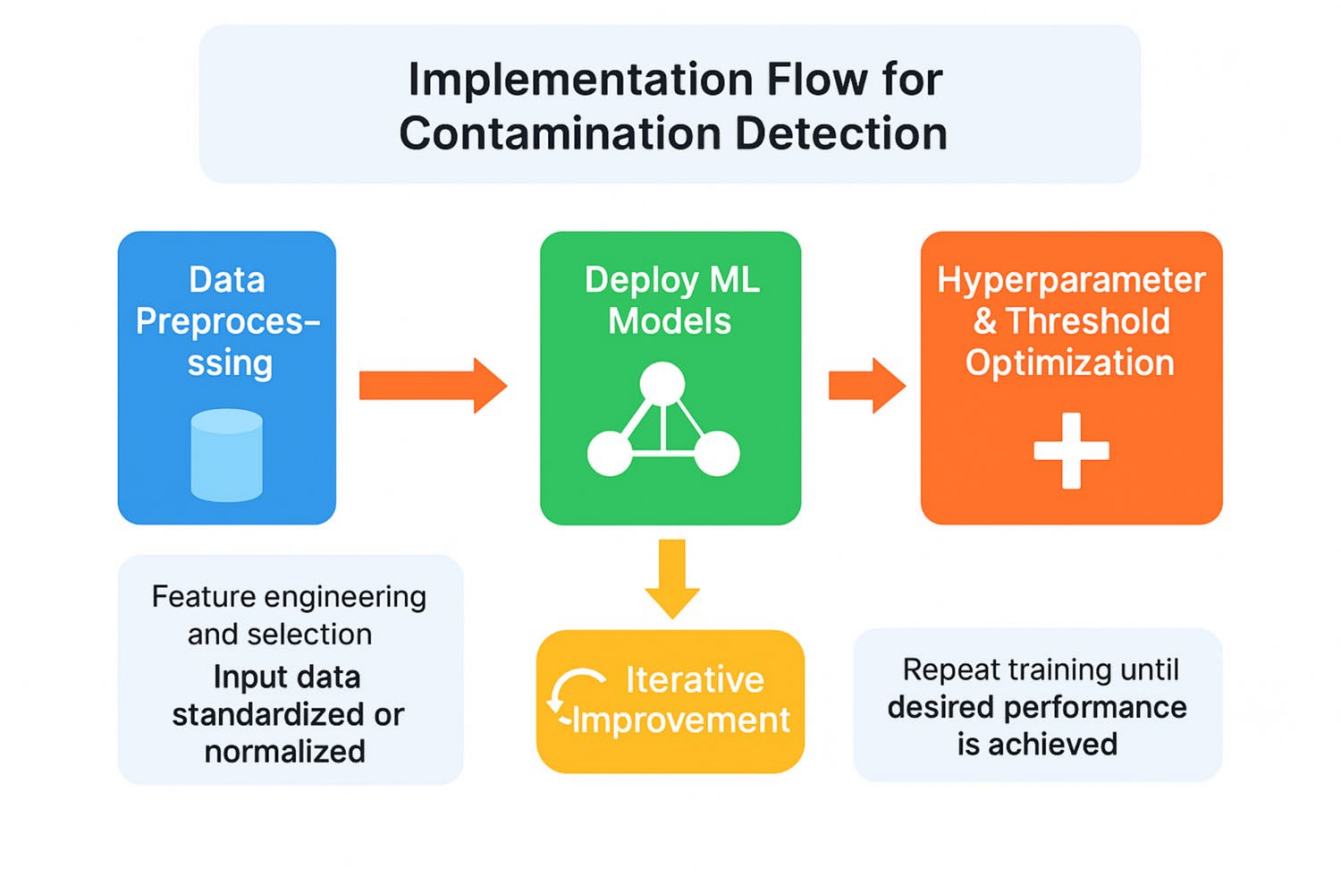
General Implementation Flowchart for Contamination Detection

Figure 1 shows the general implementation workflow for contamination detection using machine learning, highlighting key stages in data preprocessing, model training, and performance optimization through hyperparameter tuning and threshold adjustment. Interested readers should refer to Sections S1 in the Supporting Information for more detailed discussion and explanation about HPO [12–14] using BOHB algorithm [15] from Optuna [16].

### Threshold-based detection baseline

To quantify the added value of machine learning (ML) approaches over conventional industrial practices, we benchmark our OCSVM and AE models against a traditional threshold-based contamination detection method. This method, still used in many fermentation settings, typically flags contamination when key process variables fall outside of statistical control limits—most commonly set using the mean ± 3 standard deviations (3σ rule) based on historical non-contaminated batches. We choose three critical process variables—dissolved oxygen (DO), fermenter pressure (FP), and pH due to their well-known associations with contamination risk. For each, we compute the non-contaminated mean and standard deviation, then define the threshold range as [μ—3σ, μ + 3σ]. A batch was flagged as contaminated if any of the three variable values exceeds its respective threshold. This approach is simple to implement and represents current practices in many industrial system.

## Results and discussion

### Using one-class support vector machine (OCSVM)

Table 2 below lists all the hyperparameters to be tuned for our OCSVM model, which takes roughly 30 min to train using Google Colab v2-8 TPU (Tensor Processing Unit) on a computer with 64.0 GB of RAM and an Intel^®^ Core^™^ i7-5930 K CPU @ 3.50 GHz. Interested readers should refer to Sections S2 in the Supporting Information for more detailed Python codes on how to implement OCSVM model.

**Table 2:** Hyperparameters being tuned in the OCSVM model

Hyperparameter name	Hyperparameter type	Hyperparameter description	Hyperparameter search space	Best value
“nu”	Float^†^	Fraction of anomalies	LogUniform (0.001, 0.3_	0.0605
“gamma”	Float	Kernel coefficient	LogUniform (0.00001, 1)	0.6439
“kernel”	Categorical^‡^	Kernel function type	[“linear”, “poly”, “rbf”, “sigmoid”]^§^	“rbf”
“degree”	Int¶	Degree of polynomial kernel (if “kernel” = = ”poly”)	Int(2, 5)	–
“coef0”	Float	Independent term $c$ for polynomial and sigmoid kernels (if “kernel” = = ”poly” or “kernel” = = ”sigmoid”)	Uniform(0, 1)	–
“tol”	Float	Tolerance for convergence	LogUniform (0.00001, 0.1)	0.0024

^†^For floating point values

^‡^For different categorical options

§For corresponding linear, polynomial, RBF or sigmoid

¶For integer values

**Fig. 2:**
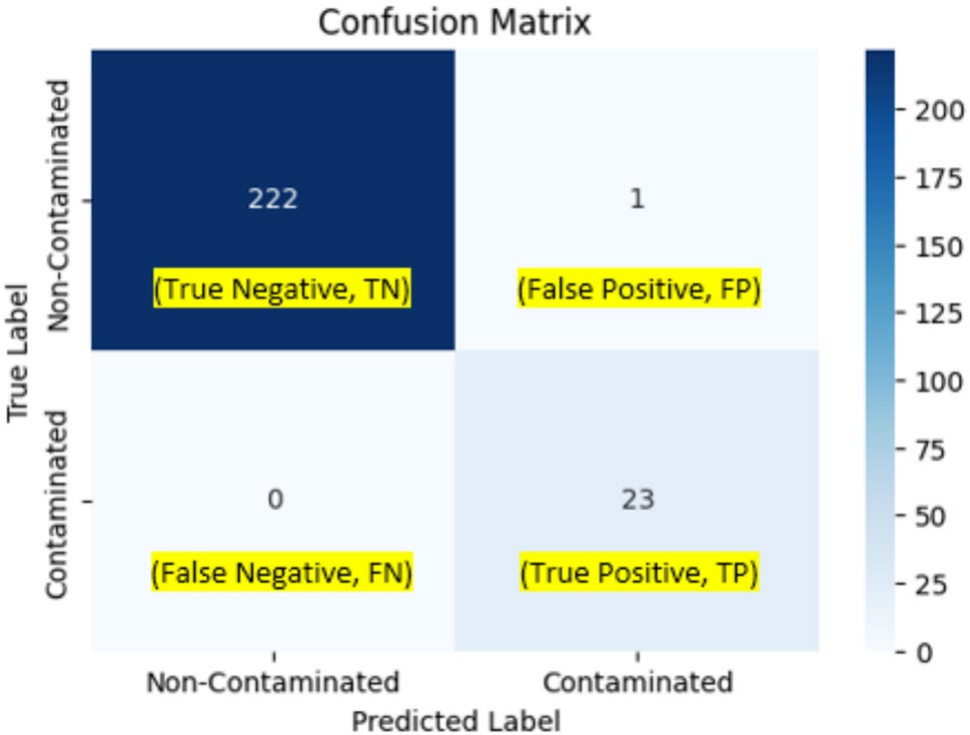
Confusion matrix for the OCSVM model

**Fig. 3:**
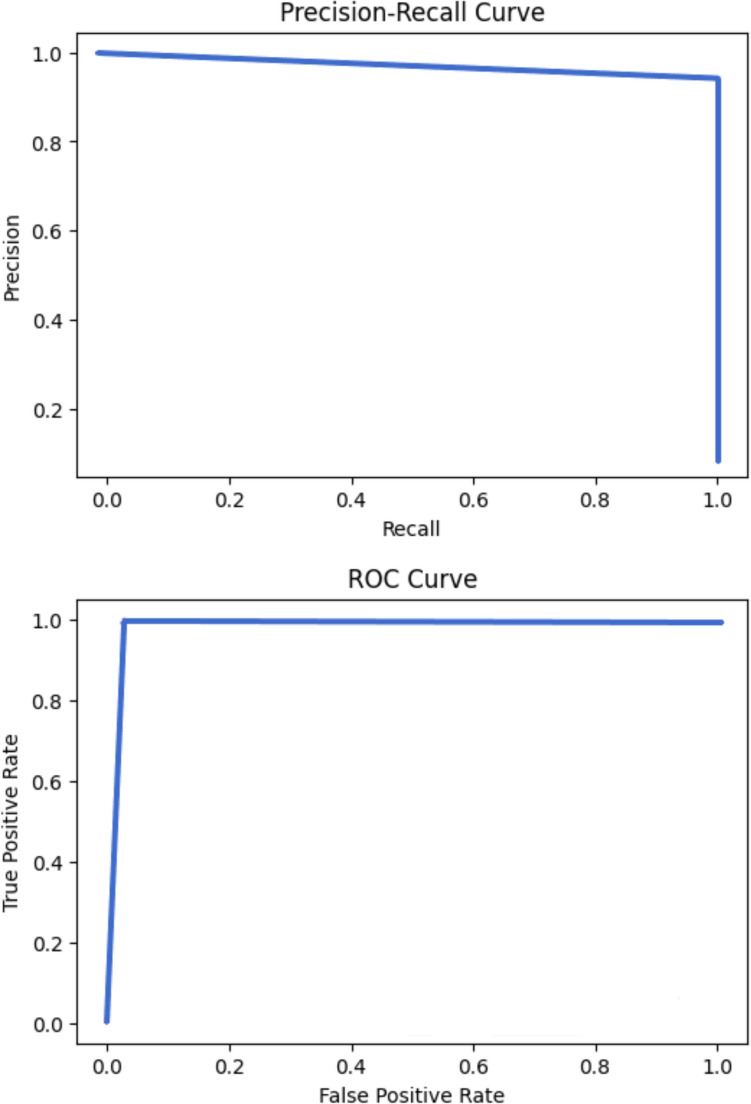
PR and ROC curves for the OCSVM model

From Table 2, we see that Optuna gives the flexibility of assigning names, types, and search space to the hyperparameters of interest with many options available, easing the HPO process and helping users to keep track of the hyperparameters. The best hyperparameter combination using the BOHB hyperparameter tuning algorithm from Optuna, resulting in the best F2-score of 0.99138, is given in the last column of Table 2. All other model performance metrics are also listed in Table 3. In addition, the confusion matrix is given in Fig. 2, and the PR and AOC plots are given in Fig. 3.

**Table 3:** Model performance metrics in the OCSVM model

Model performance metrics	Best values
Precision	0.958333
Recall	1.0
Specificity	0.995516
F2-score	0.991379
ROC-AUC	0.997758
PR-AUC	0.979167

Table 3 and Fig. 1 indicate that the OCSVM model performs exceptionally well on the task with a recall of 1.0. It correctly flags out 23 contaminated batches as true positive and correctly predicts 222 normal batches as true negative, with only one normal batch being wrongly labeled as contaminated (false negative) (hence high recall of 0.96 and precision of 1.0). From Fig. 2 and the results of AUC from Table 2, we see that the model also does very well in terms of their areas under the PR curve and the ROC curve, which are both very close to 1.

**Fig. 4:**
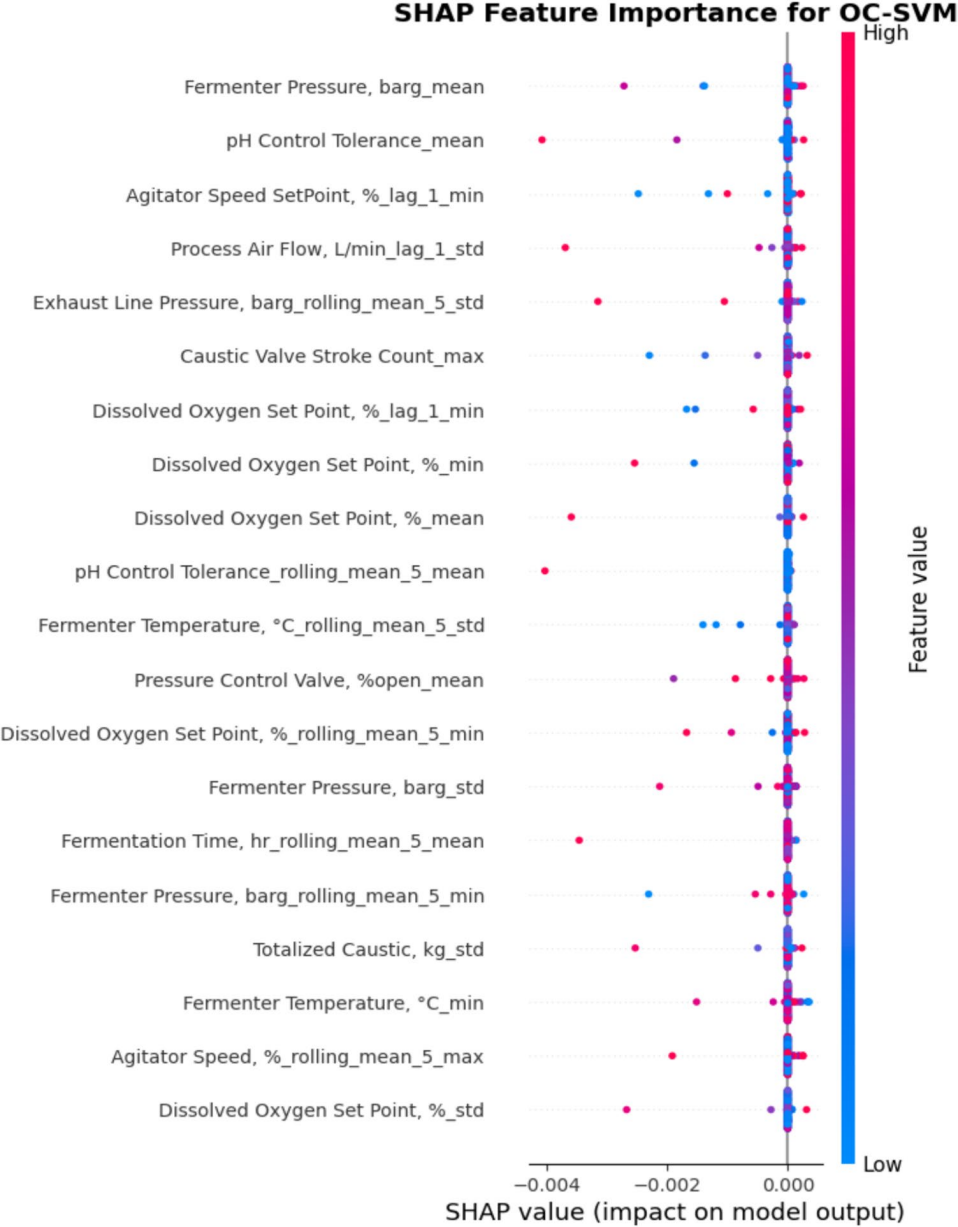
SHAP feature importance for the OSVM model

To evaluate the importance of different process variables contributing to contamination, we use Shapley Additive exPlanations (SHAP) [17]. SHAP is based on Shapley values from cooperative game theory, where each feature is treated as a “player” contributing to a model’s prediction. It is a robust, fair, and interpretable method to understand model decisions, making it a gold standard for feature importance in ML. Figure 4 plots the 20 most important features contributing to contaminations using the OCSVM model, which mainly includes dissolved oxygen (DO) setpoint, fermenter pressure (FP), fermenter temperature (FT), pH control tolerance (pHCT), agitator speed setpoint (ASS), pressure control valve (PCV), process airflow (PAF) and fermentation time.

From Fig. 4, we see that high values (red) of PCV are typically associated with negative SHAP values and vice versa, indicating that the higher the value of PCV, the less likely contaminations occur. A possible explanation is that a well-regulated PCV ensures stable fermentation conditions. When PCV remains open (high values), it indicates good pressure regulation, leading to stable conditions and lower contamination risk. On the other hand, if the PCV remains closed or fluctuates (low values), it means poor gas exchange, pressure imbalances, or process instability, which may increase contamination risk. The DO setpoint has a lot of high negative values (red in the negative direction), suggesting higher DO values tend to prevent contamination. A possible reason for this is that in fermentation, low DO can cause stress to desirable microbes and encourage the growth of unwanted contaminants, while high DO likely supports stable fermentation conditions, reducing contamination likelihood.

As for ASS, it possesses mostly negative values, suggesting that the higher the ASS, the less likely contaminations occur. Higher agitator speeds enhance mixing efficiency, ensuring even distribution of nutrients, pH, and oxygen throughout the fermentation tank, hence reducing the risk of contamination. Agitator fluctuations (observed through “lag_1” features) might indicate inconsistent mixing, which could contribute to contamination risk. FP also shows a strong influence as higher values tend to increase SHAP values, meaning a stronger correlation to contamination risk and vice versa. Fluctuations in pressure stability (indicated by rolling mean and std) contribute significantly, suggesting that abnormal pressure variations may be indicators of contamination events. Moreover, FT has a moderate influence on contamination, as higher temperature variations may be linked to contamination. The pHCT, PAF, and fermentation time, like FT, also have a moderate contribution to contamination as higher values and higher variability of those variables correlate with higher SHAP values, meaning higher contamination likelihoods.

### Using AutoEncoder (AE)

**Fig. 5:**
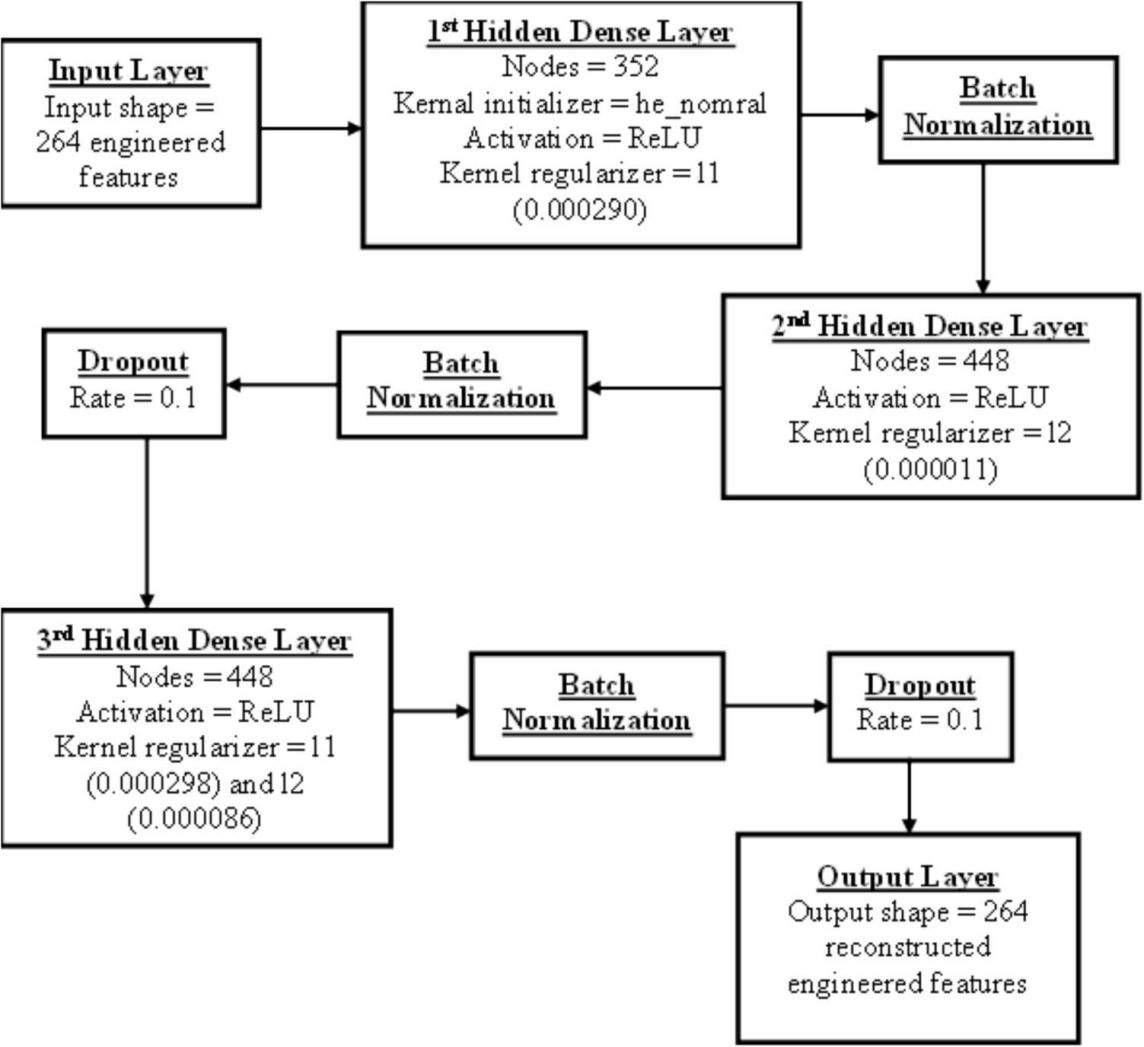
The structure of the AE after HPO

**Fig. 6:**
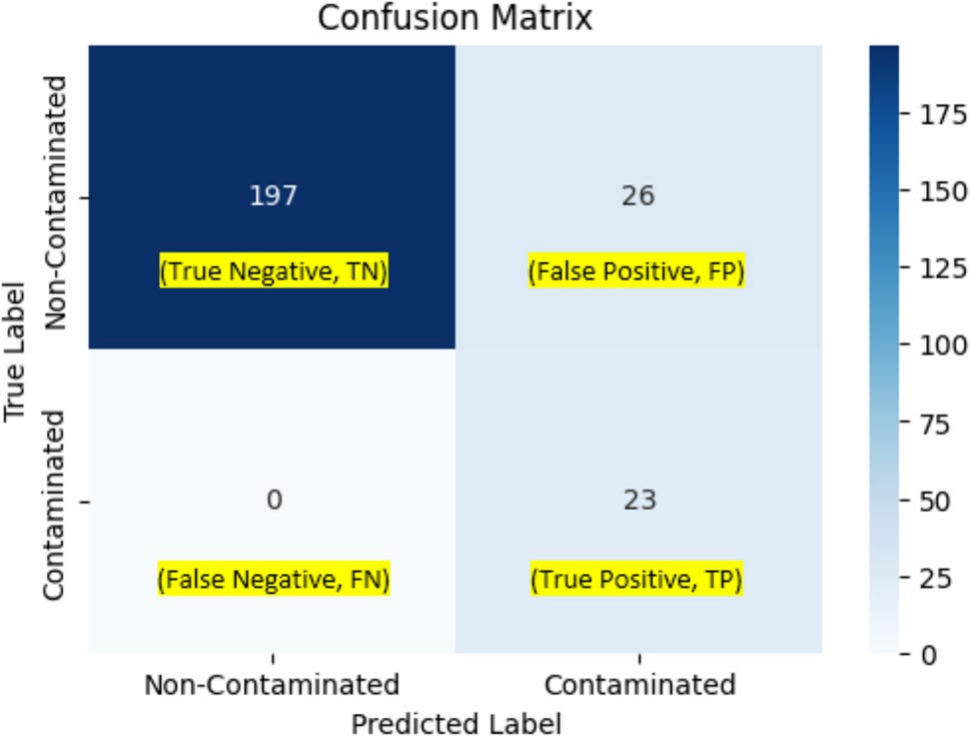
Confusion matrix for the AE model

Tables 4 and 5 list all the hyperparameters to be tuned for our AE model and the best hyperparameter combination using the BOHB hyperparameter tuning algorithm from Optuna, resulting in the best F2-score of 0.81560, respectively. The model takes roughly 8 h and 34 min to train using a Google Colab High-RAM CPU on a computer with 64.0 GB of RAM and an Intel^®^ Core^™^ i7-5930 K CPU @ 3.50 GHz. All other model performance metrics are also listed in Table 6. In addition, the structure of the AE model after HPO is given in Fig. 5 and the confusion matrix for our AE model is given in Fig. 6. Interested readers should refer to Sections S3 in the Supporting Information for more detailed Python codes on how to implement OCSVM model.

**Table 4:** Hyperparameters being tuned for the AE model

Hyperparameter name	Hyperparameter type	Hyperparameter description	Hyperparameter search space
“units_1”	Int	Number of neurons in the first dense layer	From 32 to 512 neurons with a step of 32 neurons
“l1_1”	Float	L1 kernel regularization for the first dense layer	Sampled logarithmically from 0.000001 to 0.01
“dropout_1”	Float	Dropout rate for the first dropout layer after the first dense layer	From 0.0 to 0.5 with a step of 0.1
“units_2”	Int	Number of neurons in the second dense layer	From 32 to 512 neurons with a step of 32 neurons
“l2_2”	Float	L2 kernel regularization for the second dense layer	Sampled logarithmically from 0.000001 to 0.01
“dropout_2”	Float	Dropout rate for the second dropout layer after the second dense layer	From 0.0 to 0.5 with a step of 0.1
“num_layers”	Int	Number of hidden dense layers after the second hidden dense layer	Either 1 or 2
“units_hid_i” with i^**^ is the corresponding i^th^ hidden layer after the second dense layer	Int	Number of neurons in the i^th^ hidden dense layer after the second dense layer	From 32 to 512 neurons with a step of 32 neurons
“l1_hid_i” with i is the corresponding i^th^ hidden layer after the second dense layer	Float	L1 kernel regularization for the i^th^ hidden dense layer after the second dense layer	Sampled logarithmically from 0.000001 to 0.01
“l2_hid_i” with i is the corresponding i^th^ hidden layer after the second dense layer	Float	L2 kernel regularization for the i^th^ hidden dense layer after the second dense layer	Sampled logarithmically from 0.00001 to 0.01
“dropout_hid_i” with i is the corresponding i^th^ hidden layer after the second dense layer	Float	Dropout rate for the i^th^ dropout layer after the second dense layer	From 0.0 to 0.5 with a step of 0.1
“learning_rate”	Categorical	Learning rate for the optimizer	0.0001, 0.001, or 0.01
“optimizer”	Categorical	Types of optimizers	Adam, RMSprop, or SGD
“batch_size”	Categorical	Batch size used for training	8, 16, or 32

^**^Based on our own Python code script, “i” starts from “0” for the first additional hidden dense layer from the second dense layer

**Table 5:** The best hyperparameter combination after HPO for the AE model

Hyperparameter name	Best values
“units_1”	352
“l1_1”	0.000290
“dropout_1”	0.0
“units_2”	448
“l2_2”	0.000011
“dropout_2”	0.1
“num_layers”	1
“units_hid_0”	448
“l1_hid_0”	0.000298
“l2_hid_0”	0.000086
“dropout_hid_0”	0.2
“learning_rate”	0.0001
“optimizer”	RMSprop
“batch_size”	32

**Table 6:** Model performance metrics in the AE model

Model performance metrics	Best values
Precision	0.469388
Recall	1.0
Specificity	0.88341
F2-score	0.815603
ROC-AUC	0.941704
PR-AUC	0.734694

Table 6 and Fig. 6 indicate that the AE model performs reasonably well on the task, but falls slightly behind the OCSVM model in terms of mislabeling normal batches as contaminated (hence lower in precision and PR-AUC values). However, it still correctly identifies all contaminated batches and achieves a recall of 1, which is crucial for this contamination detection task.

Unlike OCSVM, which directly predicts contamination probability or anomaly scores, the autoencoder reconstructs the input features. Understanding which features contribute to anomalies is essential for actionable contamination detection. While SHAP is a widely used model-agnostic method for explaining predictions of supervised learning models, its application to unsupervised models like AEs requires adaptation.

In supervised learning, SHAP assigns each input feature an importance value for a specific prediction, based on its contribution to the model’s output (e.g., a class probability or regression score). However, in AEs used for anomaly detection, the output is a reconstruction of the input itself, and the anomaly score is derived indirectly as the reconstruction error—typically the MSE between input and output. This poses two main challenges:

1. Lack of a direct prediction target: Unlike supervised models, the AE does not produce a single prediction target. The anomaly score is computed post-hoc from the model’s reconstruction, making it unclear how to meaningfully attribute the scalar error back to individual input features.
2. Aggregation of feature effects: The reconstruction error is an aggregate of feature-level differences, meaning it does not represent the contribution of individual features in a direct or interpretable way. Applying SHAP to this aggregate scalar can produce inconsistent or misleading attributions.

**Fig. 7:**
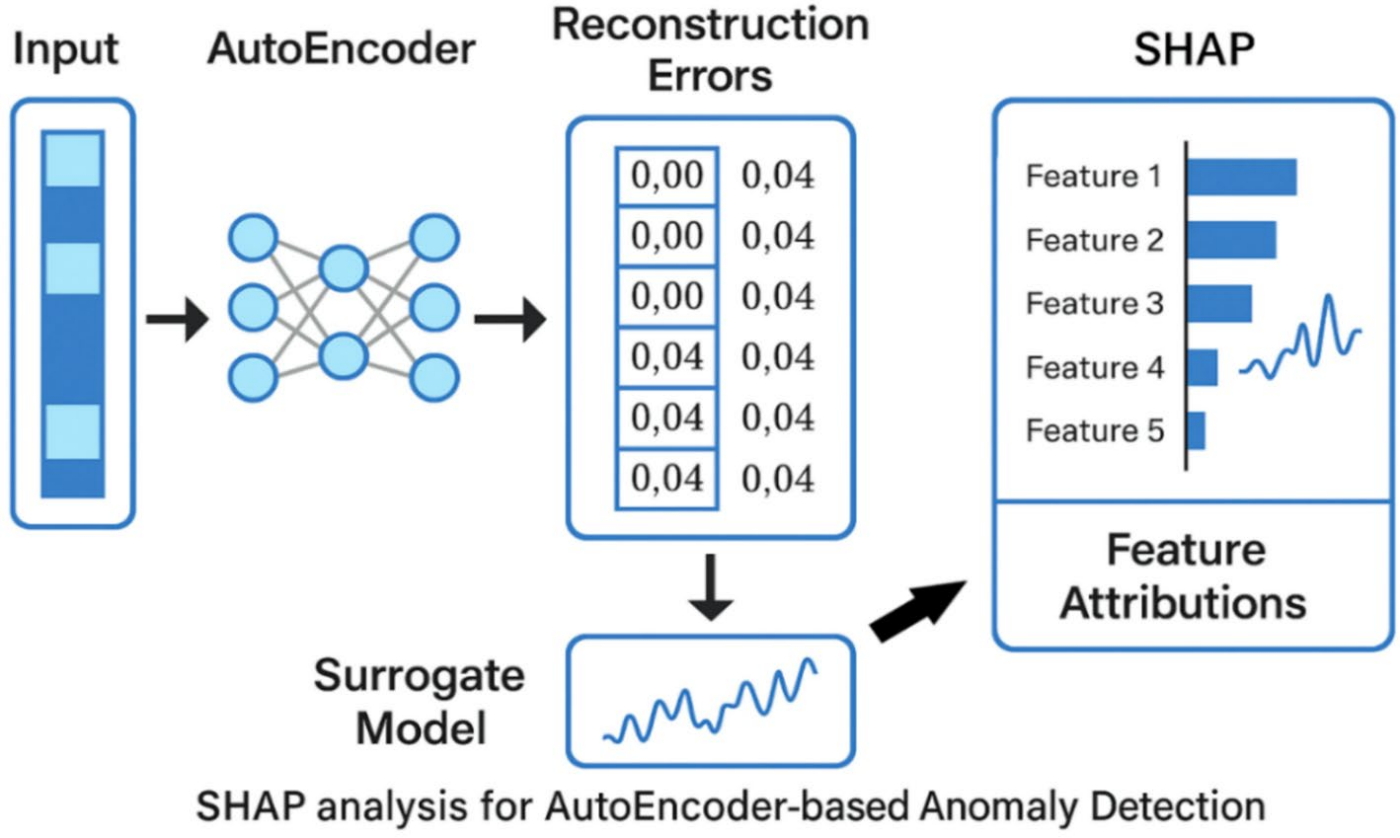
SHAP analysis for AE-based anamoly detection

To address these limitations and enable consistent interpretability, we adopt a two-step surrogate modeling approach (refer to Fig. 7 and Figures S3-8 in the Supporting Information for SHAP analysis for AE-based anamoly detection):

- Step 1: Per-feature error decomposition: Instead of analyzing the total reconstruction error, we calculate the squared error for each feature, creating a vector of feature-wise deviations. This provides a more granular view of how each input dimension contributes to the overall anomaly signal.
- Step 2: Surrogate model for SHAP analysis: We train a gradient-boosted decision tree regressor (using XGBoost) to predict the per-feature squared error vector from the original input features. SHAP values are then computed on this surrogate model, allowing us to estimate the contribution of each input feature to the observed reconstruction errors.

**Fig. 8:**
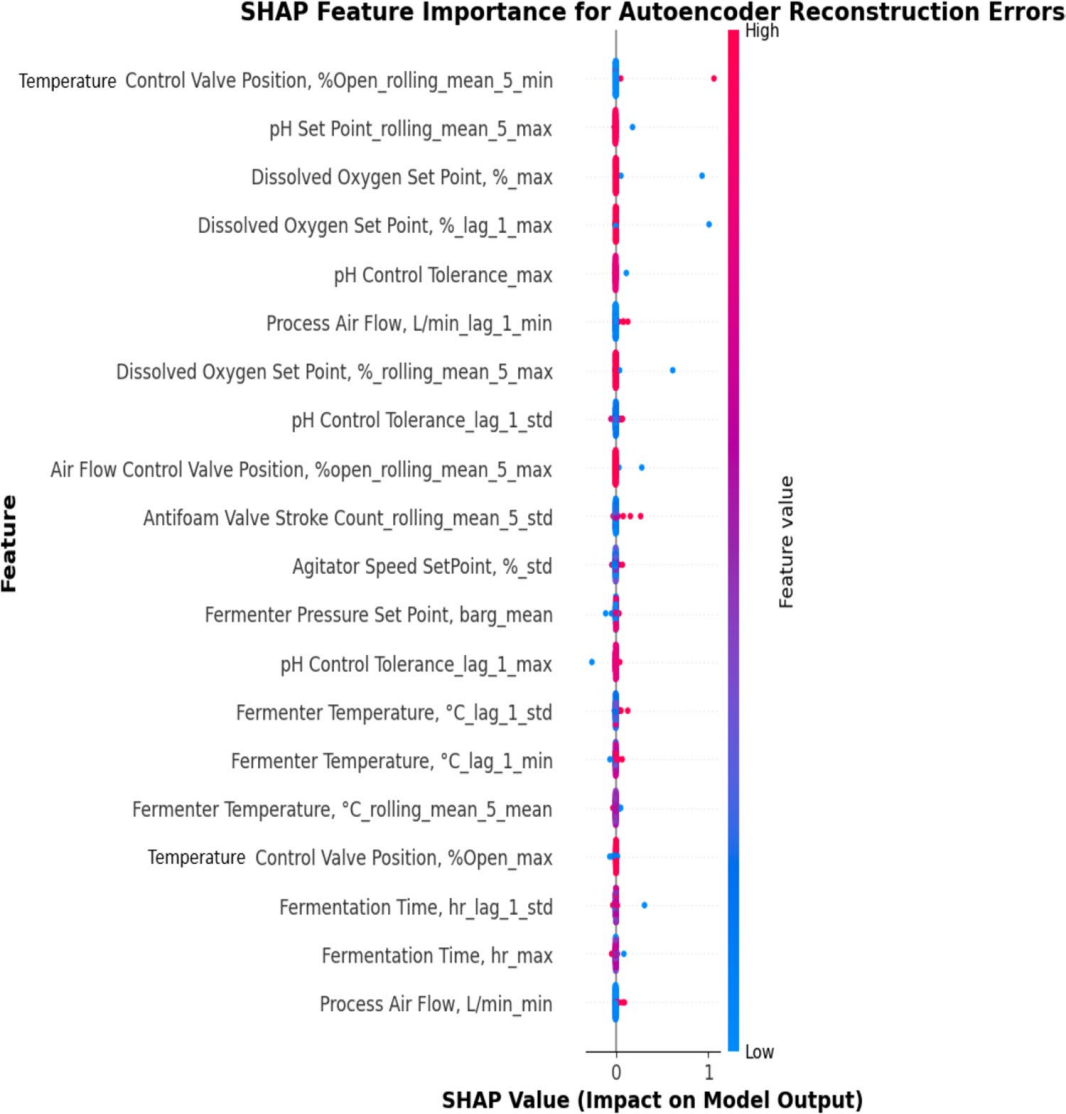
SHAP feature importance for the AE model reconstruction errors

This method yields stable and interpretable results. For example, we find that features related to oxygen uptake rate, agitation, and pH frequently contribute to elevated reconstruction errors in contaminated batches, aligning well with domain knowledge of fermentation disruptions. Figure 8 plots the 20 most important features contributing to contaminations using the AE model, which mainly includes temperature control valve position (TCVP), dissolved oxygen (DO) setpoint, pH setpoint, airflow control valve position (ACVP), process airflow (PAF) and fermentation time. There are high SHAP values for these process variables, meaning that their values are hard to construct for the AE, and their deviations are characteristic of contaminated batches. The higher their values and fluctuations, the higher the risk of contamination.

By applying SHAP to the surrogate model predicting decomposed reconstruction errors, we maintain the integrity of the SHAP framework while adapting it to the unsupervised setting. This approach not only improves the transparency of AE-based anomaly detection, but also supports root-cause investigation and feature-level diagnostics in industrial applications.

In OCSVM, SHAP values indicate how much a feature pushes the prediction toward contamination or non-contamination, while in AE, SHAP values indicate how much a feature contributes to reconstruction error, meaning even features from normal samples can have high SHAP values if they were difficult to reconstruct. Therefore, the SHAP values in AE do not balance around zero, as in OCSVM. Instead, they are mostly positive because AE only learns normal patterns, and any deviation increases errors. Moreover, in OCSVM, SHAP values are roughly symmetrical, meaning that they can be both positive and negative because those models balance contamination and non-contamination probabilities, while in AE, reconstruction errors are one-sided, which means if a feature contributes to a large reconstruction error, it only increases the contamination score. This leads to predominantly positive SHAP values.

### Comparision with a threshold-based method

Table 7 compares the performance of the 3σ threshold-based approach (as detailed in Sect. "Threshold-based detection baseline") with the proposed ML models (OCSVM and AE).

**Table 7:** Model performance metrics in the AE model

Method	Precision	Recall	Specificity	F2-score
Threshold-based (3 ${\varvec{\upsigma}}$)	0.321	0.673	0.698	0.552
OCSVM	0.958	1.0	0.996	0.991
AE	0.469	1.0	0.883	0.816

While easy to implement, the 3σ threshold method performs poorly in detecting contaminated batches:

- Low Recall (0.673): Many contamination events go undetected, making this approach unreliable in practice.
- Low Precision (0.321): Very few flagged batches are actually contaminated, indicating a high false positive rate.
- Weak F2-score (0.552): The method significantly underperforms compared to both ML approaches, particularly OCSVM.

In contrast, OCSVM and AE models—trained exclusively on non-contaminated batches—are capable of capturing multivariate, nonlinear, and temporal relationships indicative of contamination. OCSVM, in particular, achieves perfect recall (no missed contaminations) while maintaining high precision (few false positives), leading to a strong F2-score of 0.991. These results clearly demonstrate the superior detection capabilities of ML-based methods, especially for tasks where early and accurate detection of rare contamination events is essential.

### Practical deployment considerations for unsupervised ML models

While the proposed OCSVM and AE models demonstrate high performance in detecting contaminated batches under retrospective analysis, deploying these unsupervised models into real-time fermentation control systems introduces several practical challenges. These challenges must be addressed to ensure long-term robustness, interpretability, and compatibility with manufacturing constraints.

#### Concept drift and process evolution

Bioprocesses are inherently dynamic and subject to shifts over time due to variations in raw material quality, strain adaptation, equipment changes, or environmental factors. These shifts, known as concept drift, can degrade model performance if not addressed. In unsupervised learning scenarios, drift detection is challenging due to the absence of labeled feedback. However, techniques such as monitoring the distribution of reconstruction errors (in AEncoders) or decision function scores (in OCSVMs) can serve as early indicators of drift. For practical deployment, we recommend implementing routine drift analysis using statistical metrics such as the Population Stability Index (PSI) [18], Kullback–Leibler divergence [19], or two-sample Kolmogorov–Smirnov tests [20] to detect distributional changes in model inputs or outputs. If significant drift is detected, model retraining or revalidation can be triggered automatically.

#### Retraining strategy and model maintenance

Both OCSVM and AE models can be retrained periodically using new batches confirmed to be non-contaminated. Because these models do not require contamination labels for training, they are well suited for industrial settings where contamination events are rare and difficult to label. We can use a rolling or sliding-window approach to update the training set, preserving recent operational context while discarding outdated data. To reduce operational burden, we can schedule the retraining during routine process shutdowns or integrated into a continuous validation pipeline. Where possible, incremental learning strategies may be adopted to avoid full retraining from scratch. For example, autoencoders can be fine-tuned using transfer learning on recent data while freezing earlier layers to retain general patterns.

#### Inference latency and system integration

Inference latency is a critical constraint in automated control systems, particularly for batch monitoring. Fortunately, once trained, both OCSVM and AE models exhibit low computational overhead. In our experiments, prediction time per batch was under 100 ms using standard CPU hardware. This allows for integration with industrial data acquisition systems and distributed control systems (DCS) without real-time bottlenecks. The models can be deployed as part of soft sensor frameworks, where predictions are updated at key time points in the fermentation cycle or at the end of each batch. In addition, the interpretability of OCSVM decision scores and AE reconstruction errors can be visualized on process dashboards, allowing process engineers to trace anomaly signals back to root-cause variables using SHAP-based feature attribution.

#### Model validation, regulatory considerations, and change management

For regulated environments, such as pharmaceutical or food biotechnology, any deployment of ML models requires validation under Good Manufacturing Practice (GMP) guidelines [21]. The unsupervised nature of the proposed models simplifies documentation by reducing the reliance on contaminated ground truth—which are actual, and verified labels (whether it is contaminated or not), but it also necessitates robust procedures for performance monitoring, alert management, and retraining justification. We recommend documenting model behavior under representative normal conditions, defining thresholds for anomaly flagging, and validating these thresholds against historical contamination events where possible. Change control procedures should be in place to govern retraining frequency, model versioning, and post-deployment drift detection alerts.

In summary, while unsupervised models such as OCSVM and AE offer powerful tools for contamination detection in data-limited environments, careful planning and infrastructure support are needed to handle drift, retraining, latency, validation, and compliance. These practical considerations are essential to realizing the full value of ML-driven monitoring in industrial fermentation processes.

## Future work and broader model benchmarking

Building on the current study’s demonstration of AE and One-Class Support Vector Machine (OCSVM) for contamination detection, we suggest several avenues for future research to extend the robustness, generalizability, and deployment potential of anomaly detection frameworks in bioprocessing systems.

### Expansion of anomaly detection models

The current study focused on AE and OCSVM as representative models from two major categories: reconstruction-based and boundary-based anomaly detection. To enrich comparative analysis and evaluate the full potential of unsupervised learning strategies, we propose to incorporate a broader range of models in future work. We encourage the readers to refer to the cited references for more detailed theoretical background.

- Isolation Forest (IF) [22]: an ensemble-based method that isolates outliers through recursive feature partitioning. Its scalability and independence from distribution assumptions make it a valuable addition for high-dimensional fermentation datasets.
- Local Outlier Factor (LOF) [23]: a density-based model that identifies local deviation in density for detecting cluster-sensitive anomalies.
- Robust Covariance Estimation (RCE) [24]: useful for identifying multivariate Gaussian anomalies in process conditions.
- Deep Support Vector Data Description (Deep SVDD) [25]: combines feature extraction via deep networks with hypersphere-enclosing optimization, generalizing the concept of OCSVM with end-to-end learning.

Each of these models will be evaluated under consistent preprocessing, contamination labeling, threshold optimization, and performance metrics, including Precision, Recall, F2-score, Specificity, and AUC, to ensure methodological rigor. Table 8 below summarizes the performance of some of the aforementioned anomaly detection models, including IF, LOF and RCE using Elliptic Envelope method [24] using the same fermentation dataset. Interested readers should refer to Sections S4 to S7 in the Supporting Information for more detaild step-by-step implementation of these methods in Python. Table 8 shows a clear dominance of our OCSVM and AE models compared to other anamoly predictions in terms of performance. IF shows very high recall but fails in precision and specificity, making it less practical while LOF, RCE, (with Elliptic Envelope method) and Deep SVDD are reasonable classical baselines and much better than IF.

**Table 8:** Performance metrics for other anomaly detection models compared to OCSVM and AE

Methods	Performance metrics			
	Precision	Recall	Specificity	F2-score
IF	0.098	1.0	0.049	0.352
LOF	0.312	1.0	0.756	0.694
RCE using Elliptic Envelope method	0.310	1.0	0.754	0.692
Deep SVDD	0.333	1.0	0.911	0.714
OCSVM	0.958	1.0	0.996	0.991
AE	0.469	1.0	0.883	0.816

### Incorporation of temporal features and online learning

Fermentation processes are inherently dynamic. Future work will incorporate temporal embeddings, lag features, and sequence models such as Long Short-Term Memory (LSTM) networks [26] or Temporal Convolutional Networks [27] to better capture time-based anomalies. In addition, the feasibility of online learning methods or sliding-window anomaly detection will be explored to support real-time deployment.

### Practical deployment considerations

Further investigation will address real-world implementation factors such as:

- Concept drift detection using techniques like PSI and Kullback–Leibler divergence;
- Model re-calibration frequency, balancing robustness with minimal retraining;
- Inference latency and computational overhead in edge-device or industrial DCS settings;
- Human-in-the-loop validation, particularly during transitional operating conditions or during abnormal fermentation startups.

### Interpretability and feature attribution across models

We also plan to extend SHAP-based interpretability to other anomaly detection models using surrogate regression techniques. This enables cross-model comparison of variable importance while enhancing trust and transparency in predictive tools.

Together, these additions aim to evolve the current pipeline into a comprehensive, industrially deployable anomaly detection framework tailored to the stringent demands of biopharmaceutical manufacturing environments.

## Conclusion

In this paper, we have successfully demonstrated two accurate and efficient ML models for contamination detection and reduction of fermentation processes using the BOHB hyperparameter tuning algorithm from Optuna, an easy-to-use, open-source Python library. We emphasize the importance of data preprocessing before being used as inputs to our models and describe in detail how it is carried out. Several common performance metrics for binary classification are used, such as precision, recall, specificity, PR-AUC, and ROC-AUC. However, with the goal of this task is to identify as many contaminated samples as possible, we introduce an F2-score performance matrix and use it as an optimized objective function for HPO instead of the commonly used F1-score since F2-score takes recall as twice as important compared to precision.

We propose to use two ML models, namely, OCSVM and AE. The OCSVM performs exceptionally well with recall, precision, F2-score, and PR-AUC values of 1.0, 0.958, 0.991, and 0.979, respectively. It outperforms the AE model by a decent margin in terms of precision and specificity. Compared to traditional threshold-based methods, which achieved an F2-score of 0.552 in our test case, the ML models—particularly OCSVM—offered improved precision, robustness, and the ability to capture subtle deviations across multiple variables and time. This highlights the value of applying machine learning to complex fermentation processes. Beyond accuracy metrics, we highlight that both OCSVM and AE models offer low-latency inference, compatibility with periodic retraining, and robustness to concept drift when paired with monitoring strategies. These characteristics make them promising candidates for deployment in automated quality control systems for industrial bioprocessing.

Based on the SHAP plots of both models, we identify several important key features contributing to contamination, including dissolved oxygen, fermenter temperature and pressure, agitator speed, process airflow, pressure control valve, pH, and fermentation time. Abrupt fluctuations and deviations of these process variables are highly correlated to contamination. Prolonged fermentation time also favors the risk of contamination. A higher amount of dissolved oxygen and a higher agitator speed tends to prevent contamination. However, a higher amount of airflow could lead to higher contamination risks. Stable fermenter temperature, pressure, and pH level also help to reduce contamination risks.

## Supplementary Information

Below is the link to the electronic supplementary material.

Supplementary file1 (DOCX 772 KB)

## Data Availability

All data are available to the readers upon request.
