# Supplementary material for: Methodology for contamination detection and reduction in fermentation processes using machine learning
Source: Bioprocess Biosyst Eng. 2025 Jun 26;48(9):1547–63. doi: 10.1007/s00449-025-03194-6 (PMC12367959; doi:10.1007/s00449-025-03194-6)
Supplement: Supplementary file 1 — Supplementary file1 (DOCX 772 KB) [file 449_2025_3194_MOESM1_ESM.docx]

**Supporting Information to:**

**Methodology for contamination detection and reduction in fermentation processes using machine learning**

Xuan Dung James Nguyen^1^, Y. A. Liu^1,^*, Christopher C. McDowell^1,2^, Luke Dooley^2^

^1^Aspen Tech Center of Excellence in Process System Engineering,

Department of Chemical Engineering, Virginia Polytechnic Institute and State University Blacksburg, VA 24061

^2^Novonesis Biological, Inc., 5400 Corporate Circle, Salem, VA 24153

*send correspondence to: [design@vt.edu](mailto:design@vt.edu)

**S1. Hyperparameter tuning using BOHB from Optuna**

Hyperparameters are certain sets of parameters in the learning algorithms that must be set before the training or learning processes [1]. These hyperparameters are very different from model parameters that the learning algorithms learn during training, such as weights and biases in a neural network model. The process of efficiently setting all the necessary hyperparameter values, which results in the best performance on the dataset in a reasonable amount of time before the training phase, is called hyperparameter optimization (HPO) [2, 3].

In this work, HPO plays a crucial role in optimizing the performance of ML models for contamination detection in fermentation processes. As we deal with a highly imbalanced dataset, HPO allows us to reduce false negatives (FN) and false positives (FP), and maximize precision and recall. HPO also improves the detection performance of ML methods by parallelly tuning as many hyperparameters as possible, ensuring robust generalization, avoiding overfitting, and speeding up model training and deployment.

In this paper, we utilize a powerful yet user-friendly and easy-to-learn Python hyperparameter tuning package called Optuna [4], which supports an HPO algorithm called Bayesian Optimization with HyperBand (BOHB). BOHB combines a Tree-structured Parzen Estimator (TPE) sampler called *TPESampler* for Bayesian Optimization and a pruner called *HyperbandPruner* for Hyperband. Details of BOHB and all the Python codes outlining step-by-step how to deploy the BOHB algorithm from Optuna to do HPO for each ML method are given in the Supporting Information of this paper. The potential of combining Bayesian optimization and hyperband in a single algorithm called BOHB was proposed by Wang et al. [5]. The key motivation of this combination is to combine the strengths of both algorithms, Bayesian optimization, and hyperband, together. Hyperband does not utilize history information of previously explored hyperparameter configurations, and therefore, it is a very fast algorithm. Bayesian optimization, on the other hand, based on Bayes’ theorem, does not ignore history when sampling the next configurations, and therefore, it takes longer than hyperband to train. However, it is theoretically more accurate than hyperband, given a reasonable amount of training time. Therefore, both algorithms are actually complementary to each other in overcoming the weakness of each approach.

**S2. One-Class Support Vector Machine (OCSVM)**

**
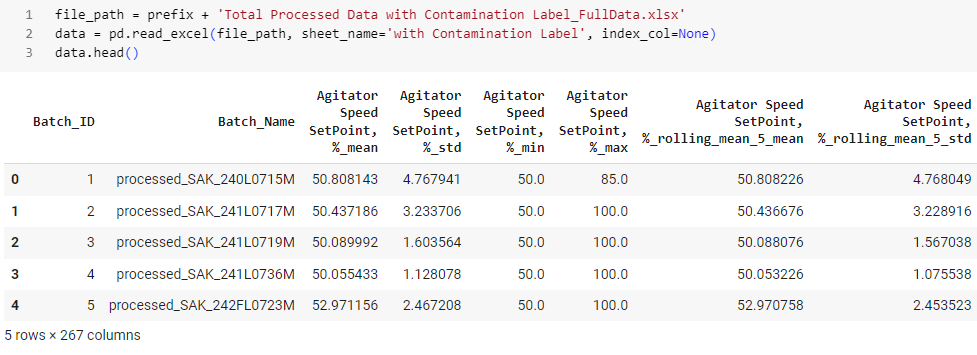
**

**Fig. S2-1** Python codes for reading the data file after preprocessing and snapshot of some of the engineered features


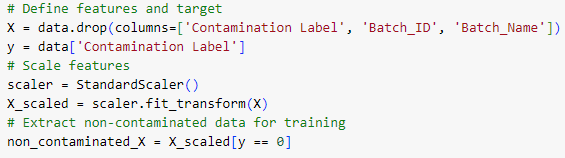


**Fig. S2-2** Python codes for getting input features and labels, standardizing features, and extracting non-contaminated data for OCSVM


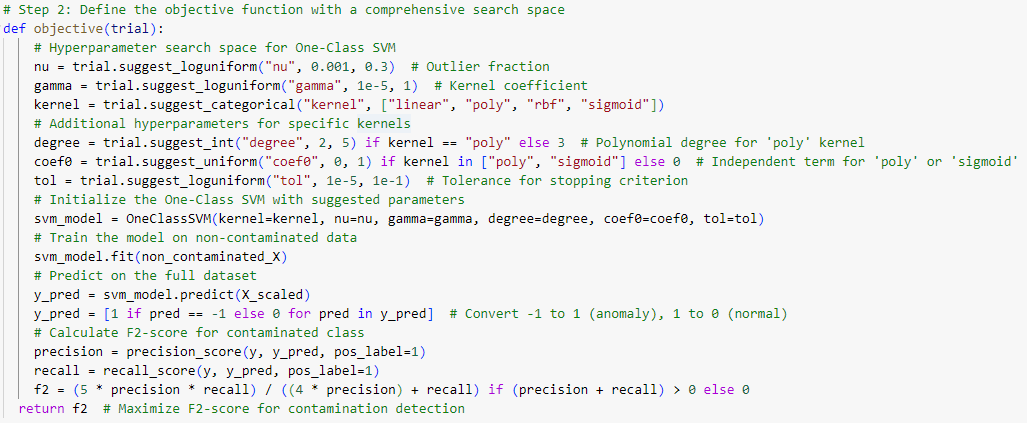


**Fig. S2-3** Python codes for defining the objective function with all the hyperparameters to be tuned and calculating the F2-score for OCSVM


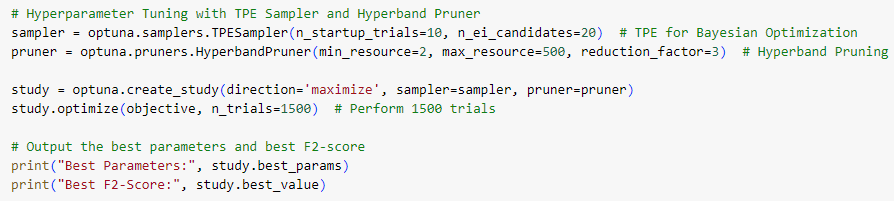


**Fig. S2-4** Python codes for setting up and running the BOHB algorithm for HPO and getting the best hyperparameter combination with the best F2-score for OCSVM


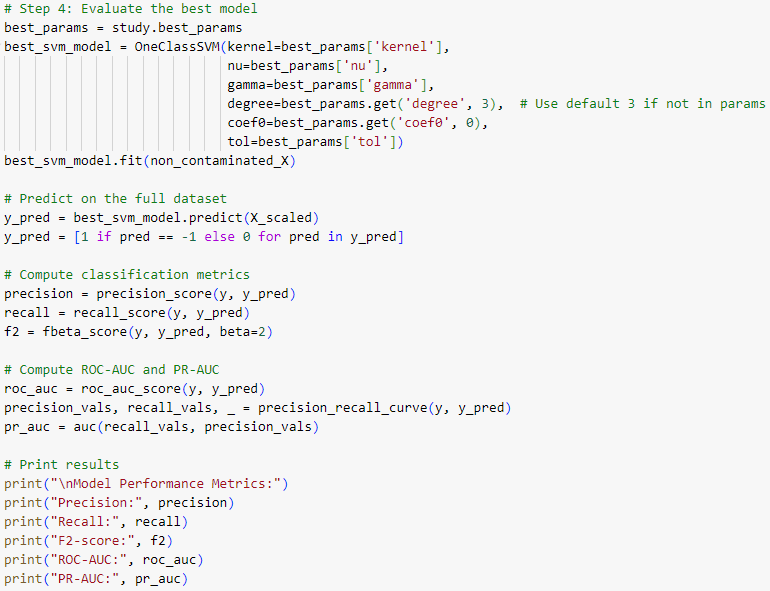


**Fig. S2-5** Python codes for evaluating the best model by fitting the best model, making predictions on the full dataset, computing and printing different classification metrics for OCSVM


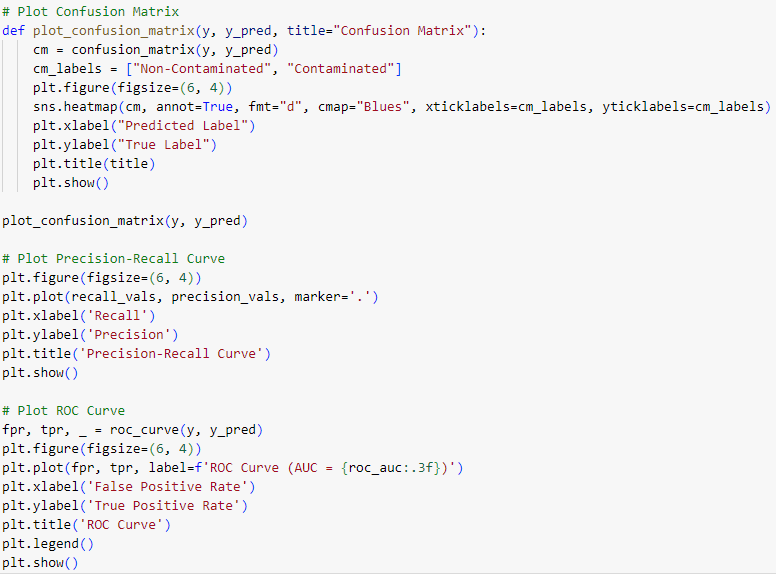


**Fig. S2-6** Python codes for plotting confusion matrix, PR, and ROC curves for OCSVM


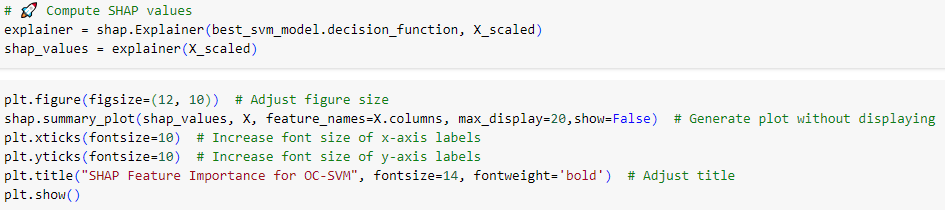


**Fig. S2-7** Python codes for computing SHAP values and plotting SHAP for OCSVM

**S3. AutoEncoder (AE)**

**
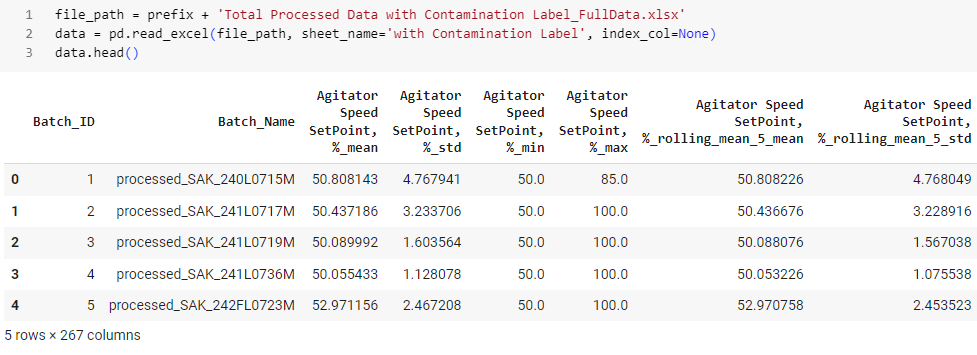
**

**Fig. S3-1** Python codes for reading the data file after preprocessing and snapshot of some of the engineered features


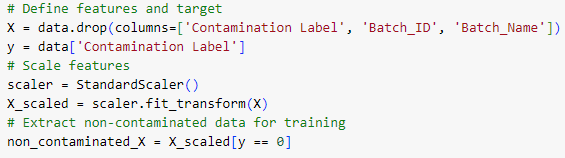


**Fig. S3-2** Python codes for getting input features and labels, standardizing features, and extracting non-contaminated data for AE


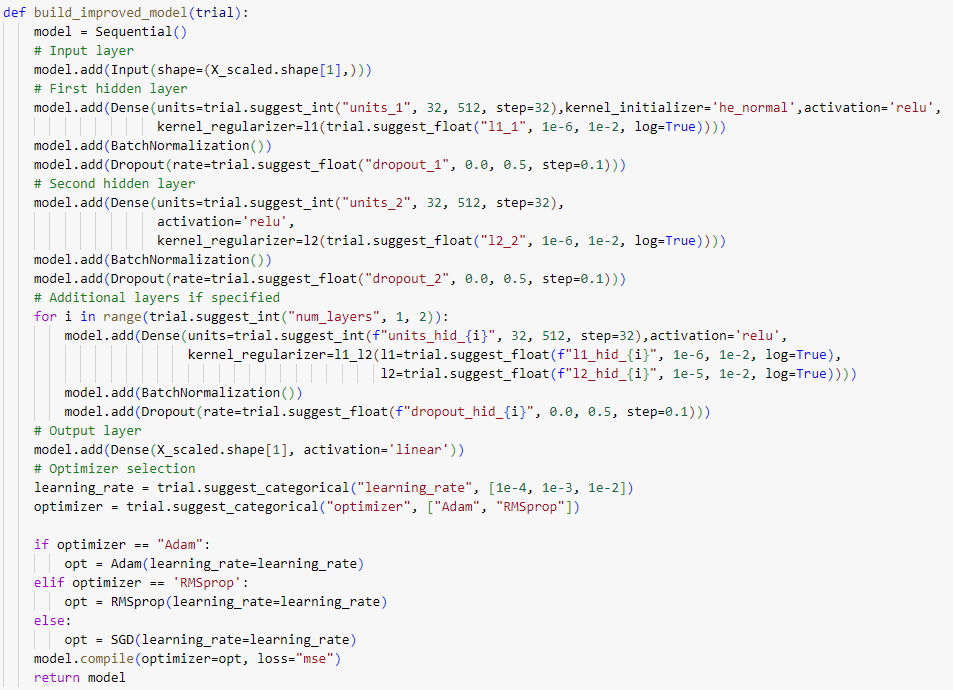


**Fig. S3-3** Python codes for defining the structure of the AE


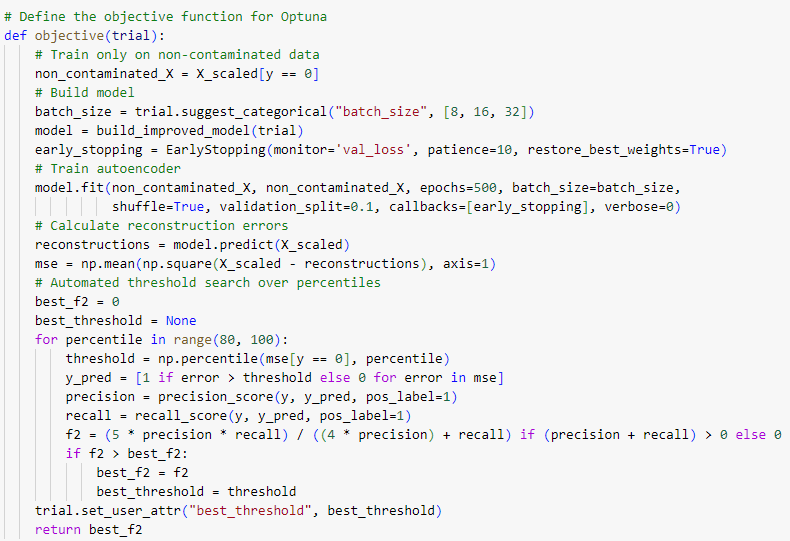


**Fig. S3-4** Python codes for defining the objective function calculating F2-score for AE


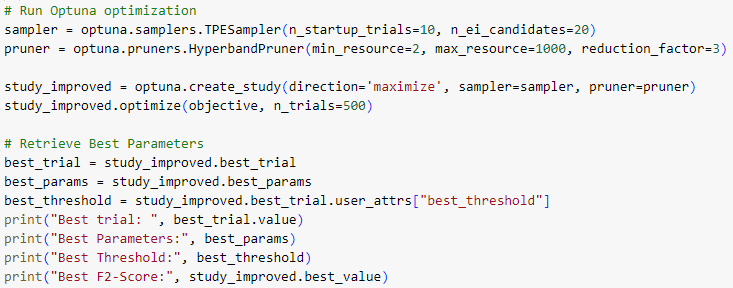


**Fig. S3-5** Python codes for setting up and running the BOHB algorithm for HPO and getting the best hyperparameter combination with the best F2-score for AE


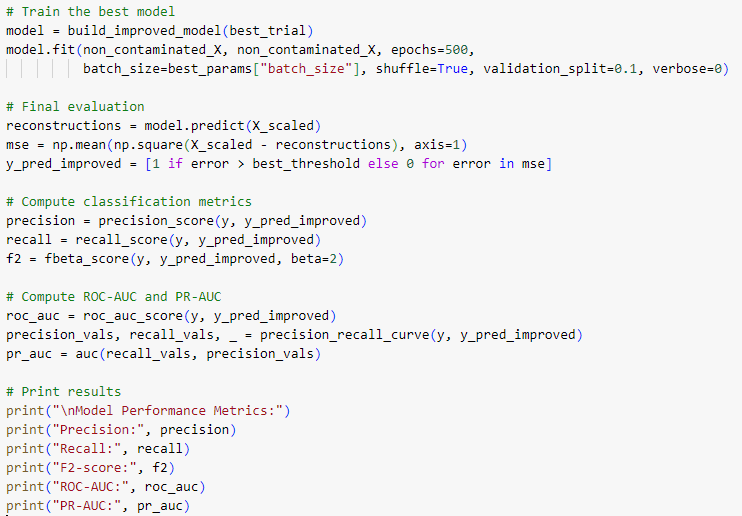


**Fig. S3-6** Python codes for evaluating the best model by fitting the best model, making predictions on the full dataset, computing and printing different classification metrics for AE


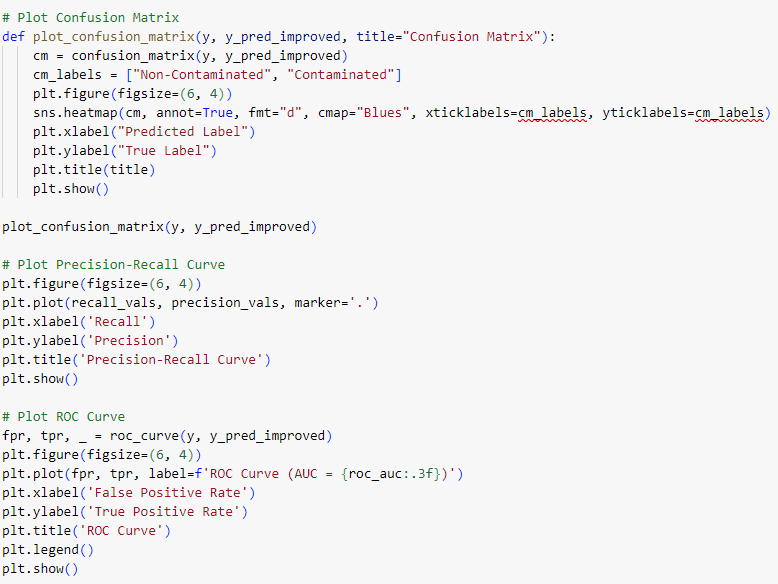


**Fig. S3-7** Python codes for plotting confusion matrix, PR, and ROC curves for AE


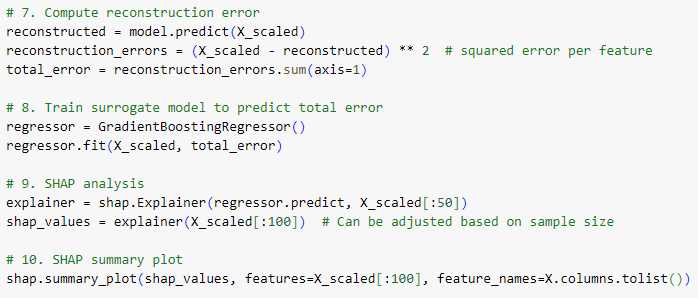


**Fig. S3-8** Python codes for the SHAP analysis for AE-based anamoly detection using surrogate model on the reconstruction errors

**S4. Isolation forest (IF)**

**
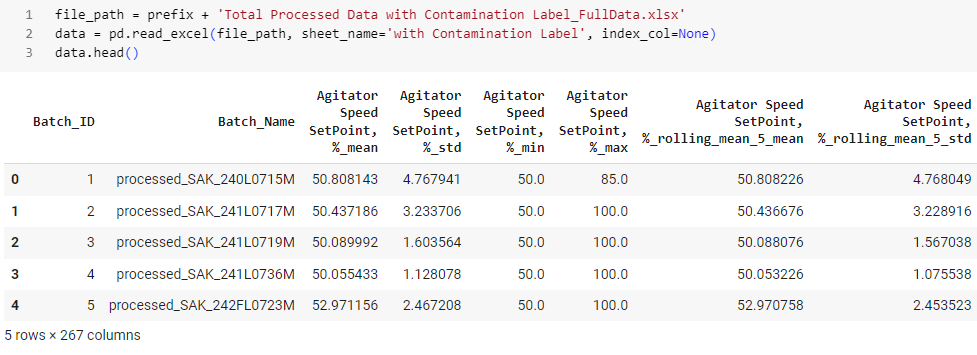
**

**Fig. S4-1** Python codes for reading the data file after preprocessing and snapshot of some of the engineered features

***
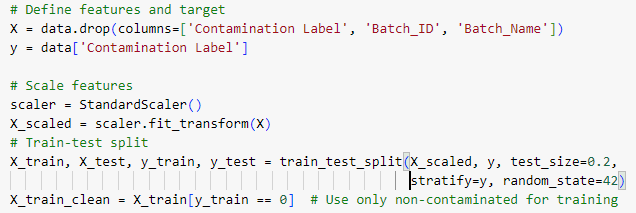
***

**Fig. S4-2** Python codes for getting input features and labels, standardizing features, train-test dataset split, and extracting non-contaminated data for training

**
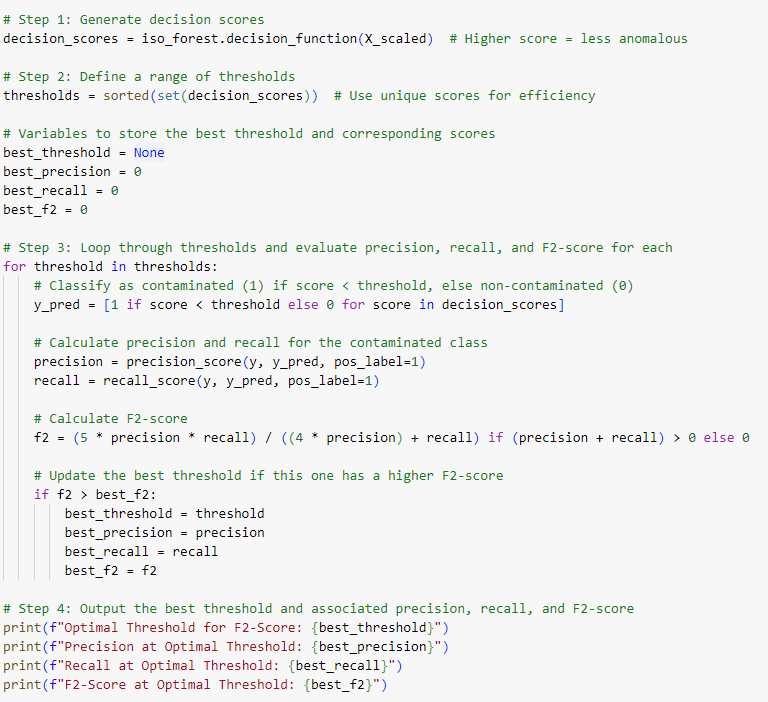
**

**(a)**

**(b)**


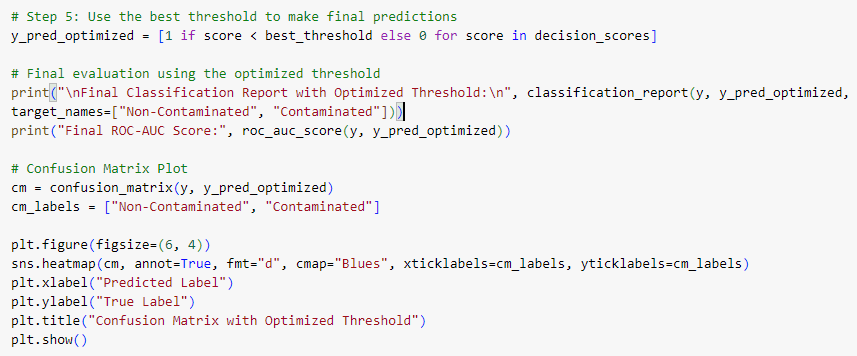


**Fig. S4-3** Python codes for IF method. (a) First part. (b) Second part.

**S5. Local Outlier Factor (IF)**

**
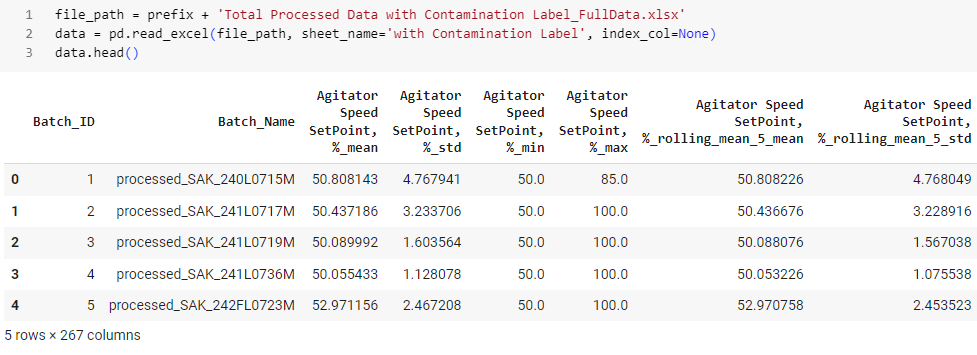
**

**Fig. S5-1** Python codes for reading the data file after preprocessing and snapshot of some of the engineered features

***
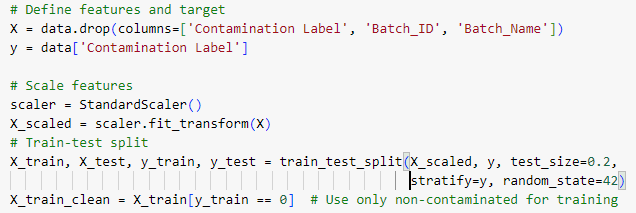
***

**Fig. S5-2** Python codes for getting input features and labels, standardizing features, train-test dataset split, and extracting non-contaminated data for training

**
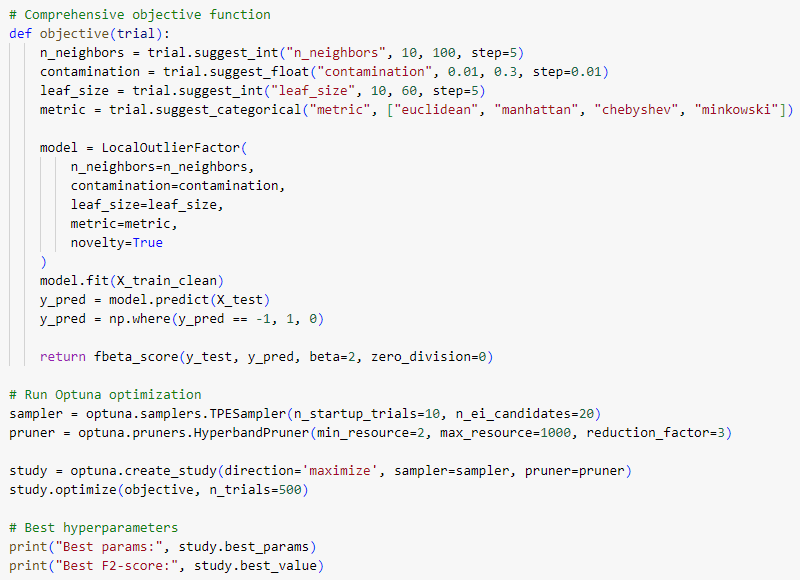
**

**(a)**

**(b)**

**
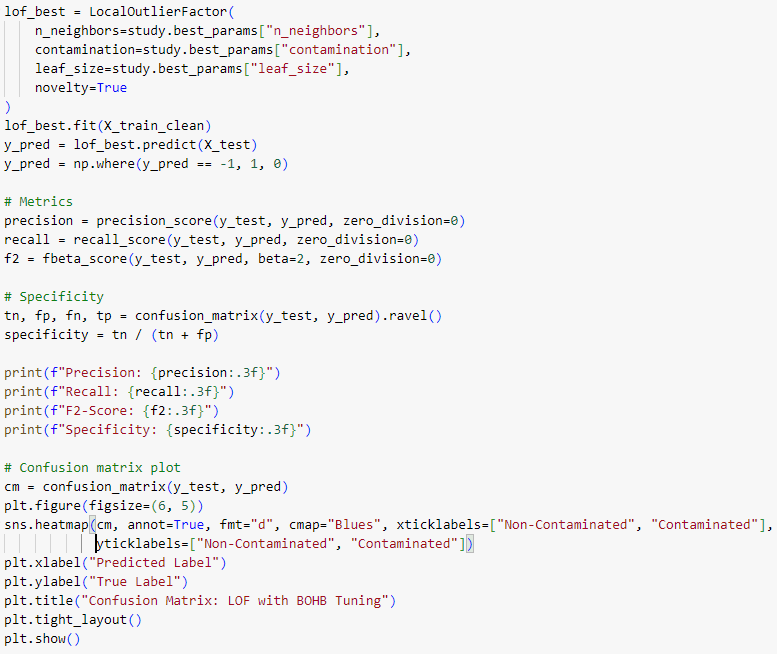
**

**Fig. S5-3** Python codes for LOF method. (a) First part. (b) Second part.

**S6. Robust Covariance Estimation (RCE)**

**
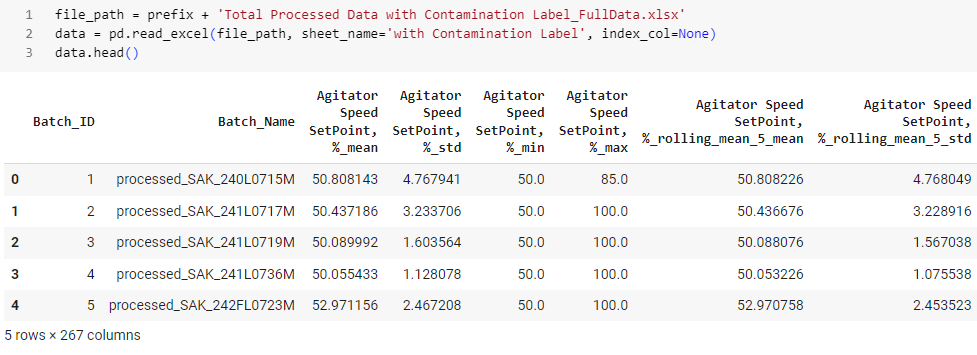
**

**Fig. S6-1** Python codes for reading the data file after preprocessing and snapshot of some of the engineered features

***
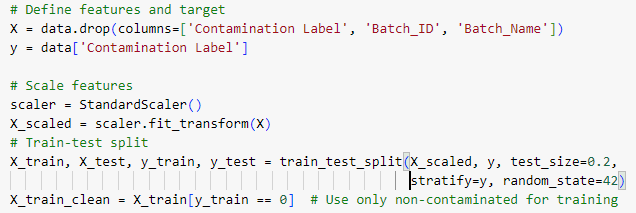
***

**Fig. S6-2** Python codes for getting input features and labels, standardizing features, train-test dataset split, and extracting non-contaminated data for training


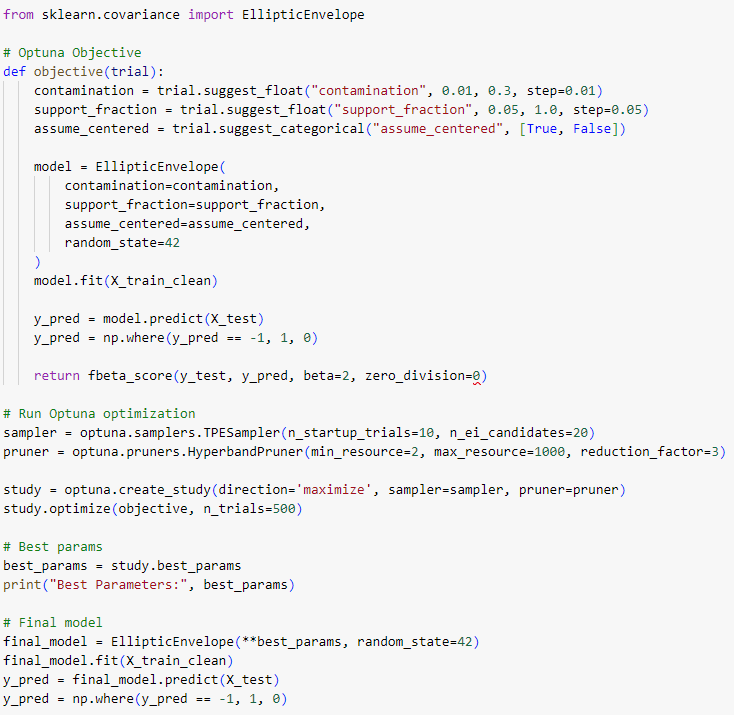


**(a)**


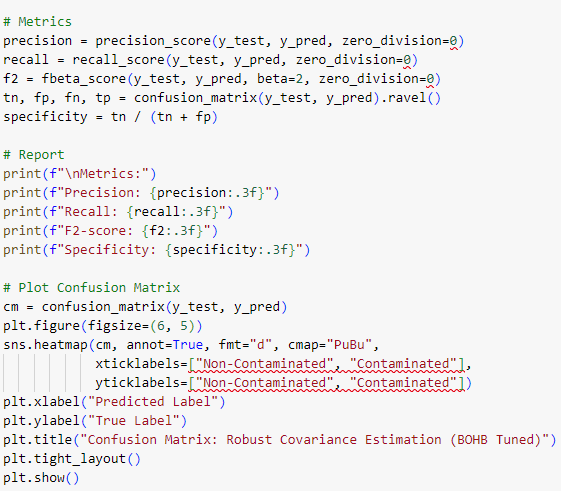


**(b)**

**Fig. S6-3** Python codes for RCE method using Elliptic Envelop. (a) First part. (b) Second part.

**S7. Deep Support Vector Data Description (Deep SVDD)**

**
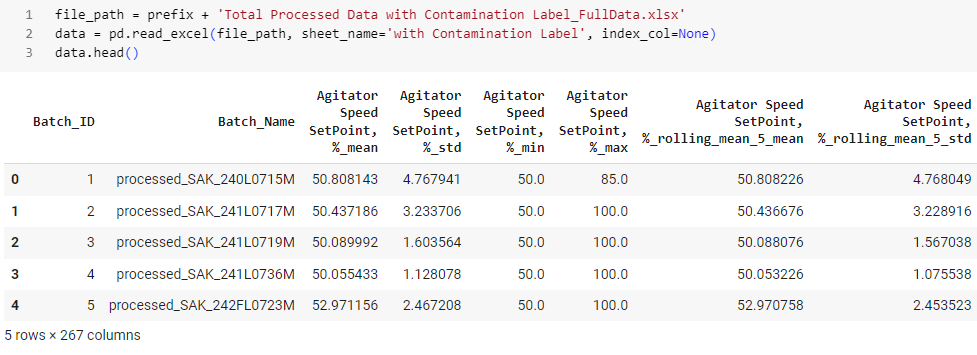
**

**Fig. S7-1** Python codes for reading the data file after preprocessing and snapshot of some of the engineered features

***
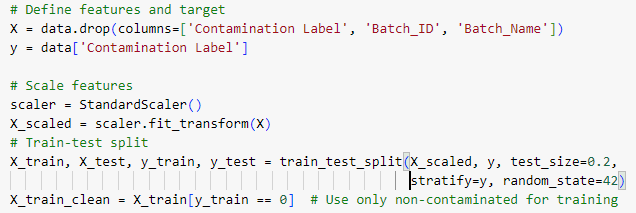
***

**Fig. S7-2** Python codes for getting input features and labels, standardizing features, train-test dataset split, and extracting non-contaminated data for training

**(a)**

***
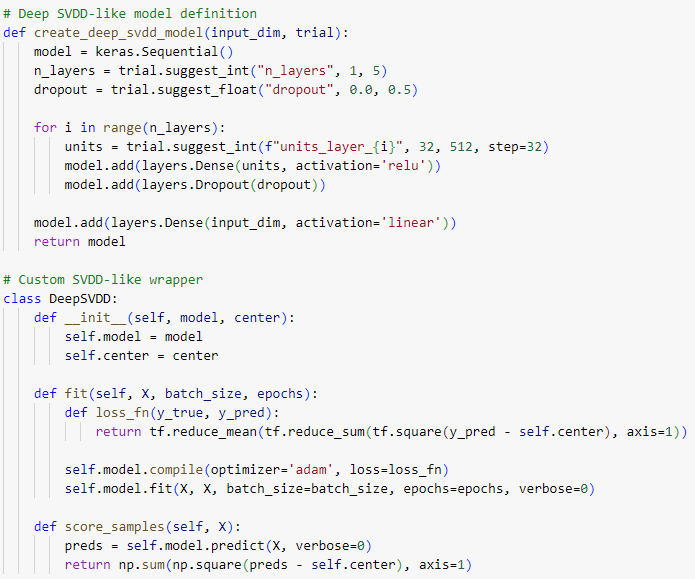
***

***
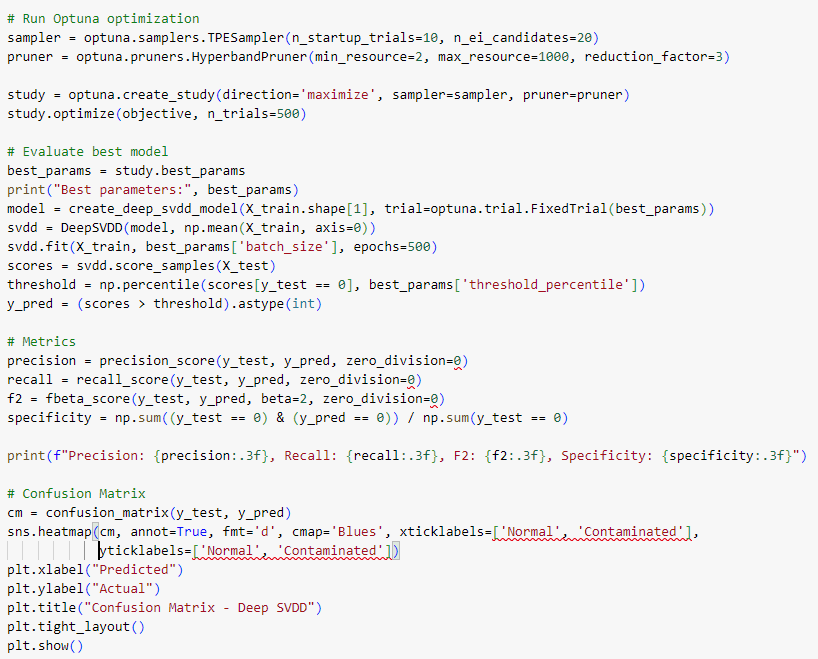
***

**(b)**

**Fig. S7-3** Python codes for Deep SVDD. (a) First part. (b) Second part.

**References**

1. Nguyen XDJ, Liu YA (2025) Methodology for hyperparameter tuning of deep neural networks for efficient and accurate molecular property prediction. Comput Chem Eng 193:108928. <https://doi.org/10.1016/j.compchemeng.2024.108928>
2. Wu J, Chen XY, Zhang H, Xiong LD, Lei H, Deng SH (2019) Hyperparameter optimization for machine learning models based on Bayesian optimization. J Electron Sci Technol 17(1):26–40. <https://doi.org/10.11989/JEST.1674-862X.80904120>
3. Yang L, Shami A (2020) On hyperparameter optimization of machine learning algorithms: Theory and practice. Neurocomputing 415:295–316. <https://doi.org/10.1016/j.neucom.2020.07.061>
4. Optuna Team (2024) Optuna: An open-source hyperparameter optimization framework. Available at: <https://optuna.org/> (accessed April 2, 2025).
5. Wang J, Xu J, Wang X (2018) Combination of hyperband and Bayesian optimization for hyperparameter optimization in deep learning. arXiv preprint arXiv:1801.01596. <https://arxiv.org/abs/1801.01596>
